# Experimental and computational studies on possibility of using glucose diazacrown cryptand as a carrier for anticancer drugs busulfan and lomustine

**DOI:** 10.1038/s41598-024-80029-6

**Published:** 2024-11-18

**Authors:** Anna Ignaczak, Marta Hoelm, Stanisław Porwański, Paweł Jóźwiak, Anna Krześlak

**Affiliations:** 1 Department of Physical Chemistry, Faculty of Chemistry, University of Lodz, 163/165 Pomorska St., 90-236 Lodz, Poland; 2 Department of Organic and Applied Chemistry, Faculty of Chemistry, University of Lodz, 12 Tamka St., 91-403 Lodz, Poland; 3 Department of Cytobiochemistry, Faculty of Biology and Environmental Protection, University of Lodz, 141/143 Pomorska St., 90-236 Lodz, Poland

**Keywords:** Drug carriers, Busulfan, Lomustine, NMR, DFT calculations, Cytotoxicity, Drug delivery, Molecular capsules, Carbohydrate chemistry, Computational chemistry, Structure prediction, Density functional theory

## Abstract

Drug carriers play a very important role in pharmacy, especially in cancer therapy. Most drugs used in the treatment of cancer are characterized by poor solubility in water and lack of selectivity in their toxic effects on normal and cancer cells. Administration of the drug in the form of a complex with an appropriately selected carrier can significantly improve its therapeutic effect and reduce side effects. In this study, the possibility of using the cryptand L1, containing two diazacrown ethers and two saccharide groups, as a potential drug carrier is investigated. In order to determine whether it can form complexes with drugs, the cryptand L1 and its complexes with two anticancer drugs, busulfan (BSF) and lomustine (CCNU), were synthesized. Their selected structural and energetic properties were investigated using both experimental and computational methods. Additionally, water solubility and cytotoxicity tests were performed for all compounds. The measured ^1^H NMR spectra confirm that L1 forms complexes L1:BSF and L1:CCNU, the solubility of which in water appears to be much higher than that of the pure drugs. The results of DFT calculations made in water described with the implicit solvent model confirm high stability of L1:BSF and L1:CCNU and indicate that L1 forms with the drugs mainly non-inclusion complexes. However, additional tests with 20 H_2_O molecules explicitly included in the model suggest that both inclusion and non-inclusion forms can occur in a real solution. Cytotoxicity studies show that the macrocycle L1 is non-toxic towards both normal and cancer cells, and its complexes with drugs show greater selectivity towards cancer cells. Interestingly, while the cytotoxicity of the L1:BSF complex is stronger than that of pure BSF, the relationship is opposite in the case of L1:CCNU and CCNU. Therefore, L1 can be considered as a potential drug carrier, especially for those drugs that have weak activity on cancer cells.

**Supplementary Information:**

The online version contains supplementary material available at 10.1038/s41598-024-80029-6.

## Introduction

The preparation of the final form of a pharmaceutical always requires taking into account a number of specific properties of a given drug. Common problems with many drugs are their poor water solubility, high toxicity, lack of cell specificity, insufficient stability, interactions with other drugs and unpleasant taste or smell. To deal with these issues, various drug delivery systems have been developed over the years^1^. Among them are the molecular drug carriers, which are capable to form a complex with a drug and transport it to the target cells^2,3^. Properly selected drug carriers can increase the solubility and stability of a drug, modify their release site and/or time, minimize unwanted side effects and prevent drug-drug interactions. A typical example of drug carriers are cyclodextrins^4^, but other molecules such as cucurbiturils, calixarenes, dendrimers or nanoparticles have also been considered^5^.

An interesting family of potential drug carriers are molecules containing crown ethers, as their presence can enhance the solubility and penetration of drugs across cell membranes, while reducing their toxicity^6^. A subgroup of such compounds are saccharide derivatives of diazacrown ethers. In the past, the properties of pseudocryptands containing two sugar units attached to one diazacrown ether via urea bridges as pendants and their ability to bind selected drugs have been investigated, using both experimental and theoretical methods^7–11^. The experimental studies have shown that such pseudocryptands form water-soluble complexes with the anticancer agent busulfan^7^ as well as with aspirin and paracetamol^8^. Extensive studies using computational methods have also been carried out to investigate at the molecular level the various properties of such pseudocryptands and the stability of their complexes with aspirin and paracetamol^9–11^. Although computational results confirmed that pseudocryptands form very stable complexes with drugs, they also showed that all of them are non-inclusion complexes in which the drug is externally attached to the pseudocryptand. This is due to the formation of hydrogen bonds between the two saccharide groups, which hold them together. More promising are new saccharide azacrown cryptands consisting of two diazacrown ethers and two saccharide units linked by urea bridges^12^. Their ability to bind drugs has not yet been investigated, while they should be able to form inclusion complexes with small drugs such as busulfan and lomustine.

Busulfan (butane-1,4-diyl dimethanesulfonate; BSF) has been one of the drugs of choice in the treatment of myeloid leukemia since the1970s. It is an alkylating agent that can produce crosslinking in DNA, which leads to the inhibition of the tumor progression. Moreover, it has been found that BSF induces DNA–protein crosslinks in human cell lines and mouse leukemia cell lines after prolonged drug treatment^13–16^. However, BSF treatment is associated with serious side effects. The small molecular weight of BSF enables it to penetrate both cancer and normal hematopoietic cells. The impact on the latter causes adverse side effects, such as chronic interstitial fibrosis or pulmonary toxicity, which is one of the most severe complications leading to acute lung injury^17–19^. Another problem is that BSF shows low cytotoxic activity against cancer cells derived from solid tumors. Kokotos et al.^20^ synthesized BSF analogs with long chains and showed that increased lipophilicity facilitated transport across the cellular membrane and increased intracellular drug accumulation. Thus, the problem in the effectiveness of the drug may be caused by difficulties in penetration across the cellular membrane.

Lomustine (1-(2-chloroethyl)-3-cyclohexyl-1-nitrosourea; CCNU) is another drug used in chemotherapy in the treatment of glioblastomas, which are the most common malignant brain tumors in adults. It is a highly lipophilic compound whose metabolites also cause alkylation and cross-linking of DNA and RNA, thus inducing cytotoxicity. As a lipid-soluble drug, it permeates the blood-brain barrier well which makes it a good candidate for the chemotherapy of brain tumors^21^. The clinical trials have shown that, when administrated in combination with other alkylating agents, procarbazine and vincristine, CCNU is the key component that improves the survival of patients. In some studies, CCNU has been used as a single agent and shown to effectively prolong overall and progression-free survival. Unfortunately, like BSF, CCNU can also cause serious side effects, such as thrombocytopenia due to cumulative bone marrow suppression, which requires the dose reduction or even discontinuation of the treatment^22–27^.

The structural properties of BSF and CCNU have been studied in the past at the molecular level by Karthick et al.^28^ and Cao et al.^29^. In both works, the search for the lowest energy conformer was carried out using DFT methods. However, all these calculations were performed in the gas phase and the configuration space was explored to a limited extent. In the case of BSF^28^ only two selected torsion angles in the molecule were varied, whereas in the case of CCNU^29^ only six arbitrary chosen structures were considered. To our knowledge, no extensive conformational search of the most stable structure of these drugs in water has been performed. Regarding their complexes with saccharide derivatives of crown ethers, there is only one experimental study mentioned above on the stability of complexes of BSF with the bis-cellobiosyl-diazacrown pseudocryptand^7^. Other candidates for BSF delivery that can possibly stabilize the drug and increase its solubility have also been considered in the literature, for example, bis-β-cyclodextrin pseudocryptand^30^, metal organic framework nanoparticles^31,32^, poly(3-hydroxybutyrate-co-3-hydroxyvalerate) polyester^33^ and carboxylatopillar^5^arene^34^. In the latter work, the complexation has been shown to improve the water solubility of BSF by a factor of 12. Also in the case of CCNU, its encapsulation into some molecules has been recently studied using either experimental or theoretical methods. Various potential carriers have been considered, such as cyclodextrins^35^, fullerenes^36^, single walled carbon nanotubes^29^ and nanoclusters^37,38^.

**Fig. 1:**
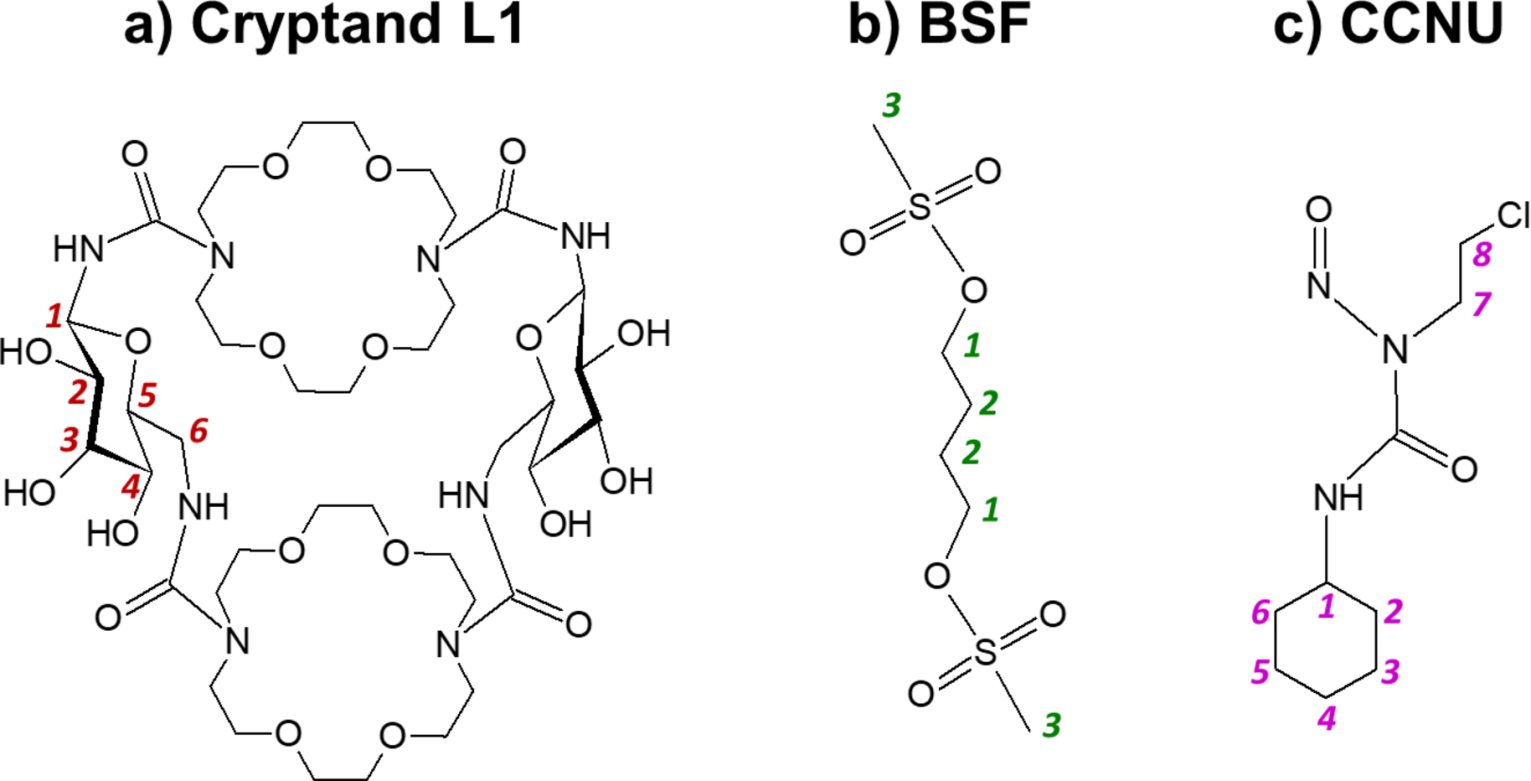
The investigated compounds and atom numbering in them: (**a**) the cryptand L1, (**b**) busulfan (BSF), (**c**) lomustine (CCNU).

In this work, we present the results of a detailed experimental and theoretical study of one of the saccharide diazacrown cryptands mentioned above, namely bis-(1,10,1′,10′)-*N*, *N*′, *N*″,*N*′′′-bis-(β-D-ureidoglucopyranosyl-4,7,13,16-tetraoxa-1,10-diazacyclooctadecane (cryptand L1; Fig. 1a), as well as its complexes with BSF (Fig. 1b) and CCNU (Fig. 1c) in stoichiometry 1:1. All experimentally prepared compounds were identified by the nuclear magnetic resonance (NMR) spectroscopy. In the theoretical approach, the Density Functional Theory (DFT) method M06-2X-GD3/6-31G(d, p) was used to indicate the energetically preferred geometries of all compounds in water and to estimate stability of the cryptand-drug complexes. Moreover, for the cryptand L1 alone, pure drugs and cryptand-drug complexes, an in vitro cytotoxicity assay was carried out against four cell lines, normal and cancer breast cells and normal and cancer colon cells.

## Experimental section

### Synthesis and spectroscopic measurement

#### General comments

All reagents and solvents were purchased from Merck and used as supplied. NMR spectra were recorded in CDCl_3_ and DMSO-d_6_, on Bruker Avance III (600 MHz for ^1^H), coupling constants are reported in Hz. The progress of the reactions was monitored by silica gel thin-layer chromatography plates (Merck TLC Silicagel60 F254). IR spectra were recorded with a Cary 630 FTIR Agilent Technologies.

#### Synthesis of the cryptand

**Fig. 2:**
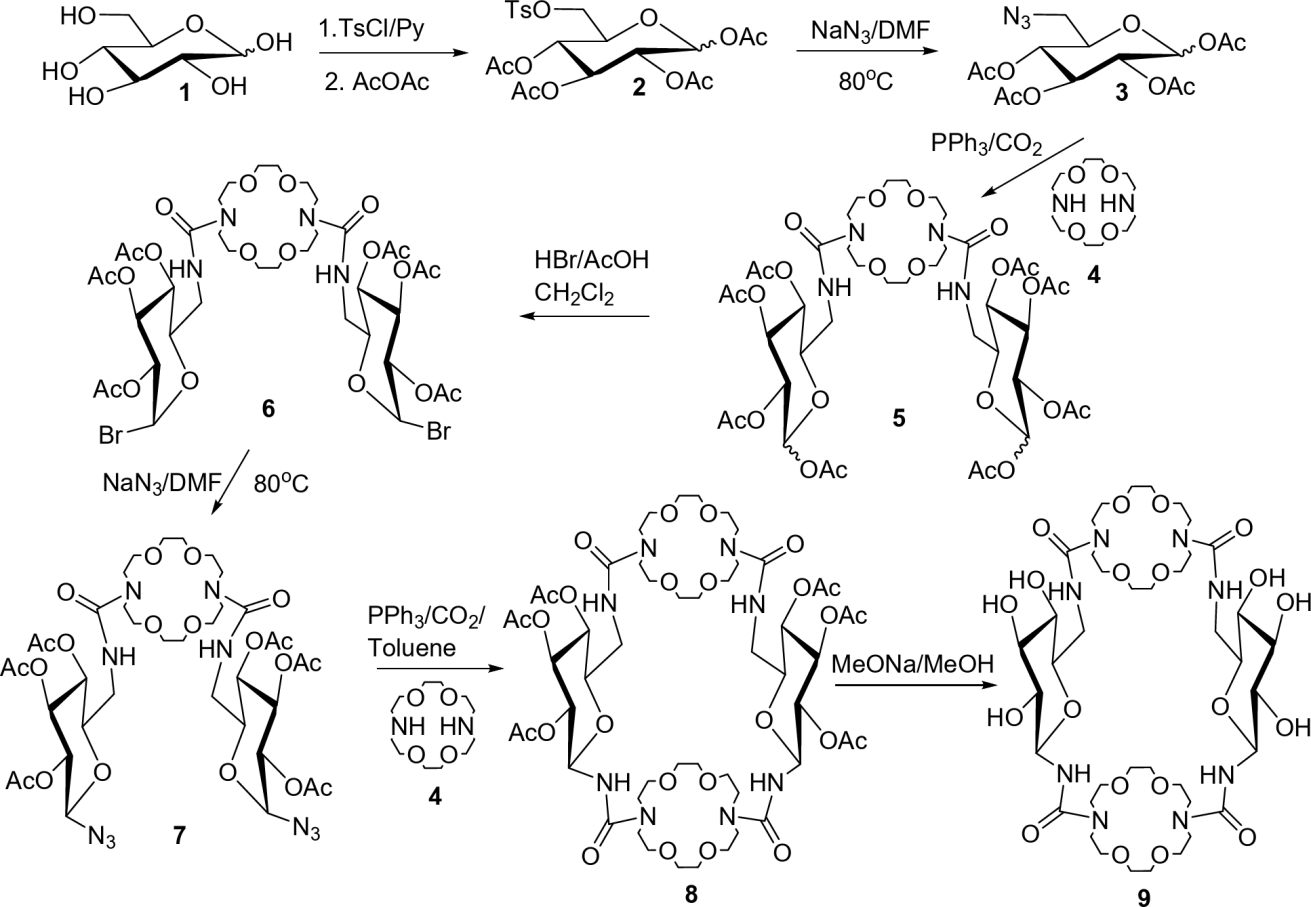
Scheme of synthesis of cryptand **9** (L1).

The cryptand **9** (L1) was obtained by multi-step synthesis (Fig. 2). The macrocycle peracetyle **8** is a compound known in the literature^12^, but the synthesis route used in that work may lead to the formation of a mixture of two different structures, in which the two disaccharide groups are either in a parallel or in an antiparallel arrangement. Since the aim of this work is to investigate the complexes of L1 with drugs, in order to obtain unambiguous results it is important to ensure that only one cryptand structure is involved in the complexation reaction. The new synthesis method presented below ensures the formation of only one product **8**, containing a parallel arrangement of carbohydrates.

The glucose **1** was reacted with tosyl chloride in pyridine to obtain predominantly 6-tosylglucose **2**. Acetic anhydride was then added to the reaction mixture. After the isolation of the product in the acetyl form **2** by column chromatography, the tosyl group was exchanged for azide with sodium azide in DMF at 80 °C. In the next step, glucose azide was subjected to the Staudinger–aza-Wittig (SAW) reaction with azacrown ether in the presence of triphenylphosphine and carbon dioxide. In product **5**, the acetyl group in anomeric position was substituted with bromine atom (product **6**), which was then replaced by an azide group (product **7**). The cryptand **8** was obtained by the SAW reaction described above. The last stage was the deprotection of hydroxyl groups using the Zemplen method. The new synthetic method allows obtaining the cryptand L1 with a yield of 16%, which is similar to that achieved with the previous method (18%) described in ref. 12. The detailed information on the synthesis of compounds **2–9** and their identification by the ^1^H NMR and/or IR spectra is provided in the Supplementary Information.

#### Complexation of BSF and CCNU with cryptand L1

The complexes were prepared according to the procedure described in a previous publication for the complex of another cryptand with BSF^7^. A solution of 2.5 mg (0.01 mmol) BSF in 0.25 ml DMSO was added under argon to a solution of 9.8 mg (0.01 mmol) cryptand **9** (L1) in 12.5 ml water at room temperature. The mixture was stirred for 18 h, which was then lyophilized to yield a quantitatively glassy product. The water solubility of the pure cryptand L1, both drugs and their complexes was estimated approximately using a simple method. For each compound separately, 1 mg of the substance was placed in a vial and water was added to it in portions of 0.1 ml. After each addition, the mixture was stirred for 10 min, and the clarity of the solution was checked with a magnifying glass. Stirring was stopped when the solution became completely clear.

### Computational details

#### Conformational search for free molecules

In order to find low energy conformers of the compounds involved in the complex formation, for each molecular system the configurational search was performed. The search was performed using the “hierarchical approach” in which the accuracy of the theoretical methods was gradually increased. In the case of substrates, a large number of different conformers were first generated in the Hyperchem program^39^ by changing the selected torsion angles (Supplementary Fig. S17). Then, the PM7 semiempirical optimizations and simulations in vacuo were carried out using the programs MOPAC^40^ and Gabedit^41^. Finally, DFT calculations were performed with the M06-2X-GD3 method^42,43^ using the Gaussian 09 package^44^. This approach has been successfully used in our earlier works on the saccharide diazacrown pseudocrytands and their complexes with selected drugs^9–11^, on the complexes of β-CD and its derivatives with some drugs^45–48^ as well as in our recent study on the interaction of ponatinib with dsDNA^49^. The choice of the M06-2X-GD3 functional was based on the tests of Boese^50^ for hydrogen-bonded molecules and on the results of a recent study of one of us on the complex of mianserin with dimethyl-β-cyclodextrin (DM-β-CD) in aqueous solution^47^. In the work of Boese, the performance of various DFT methods was investigated for the set of 49 hydrogen-bonded systems. Several functionals, such as M05-GD3, M06-GD3, M06-2X-GD3, ωB97XD, mPW1PW91 and M11, have been shown to yield small root-mean-square errors with respect to the CCSD(T)/CBS reference values. These functionals were chosen for comparative tests performed in ref. 47 for different conformers of DM-β-CD in water. The results obtained with the M06-2X-GD3 method (relative energies of ten structures and their trends) were found to be consistent with those obtained with the five other functionals, giving more reliable results than the commonly used B3LYP-GD2 functional. The M06-2X-GD3 method was also used in our recent studies of various complexes containing hydrogen bonds^48,49,51^. Due to the relatively large size of the systems, a rather modest split-valence double-zeta basis set 6-31G(d, p) containing polarization functions was used in the DFT calculations. The calculations were performed in water described by the Polarizable Continuum Model (PCM) of solvent^52^.

**Fig. 3:**
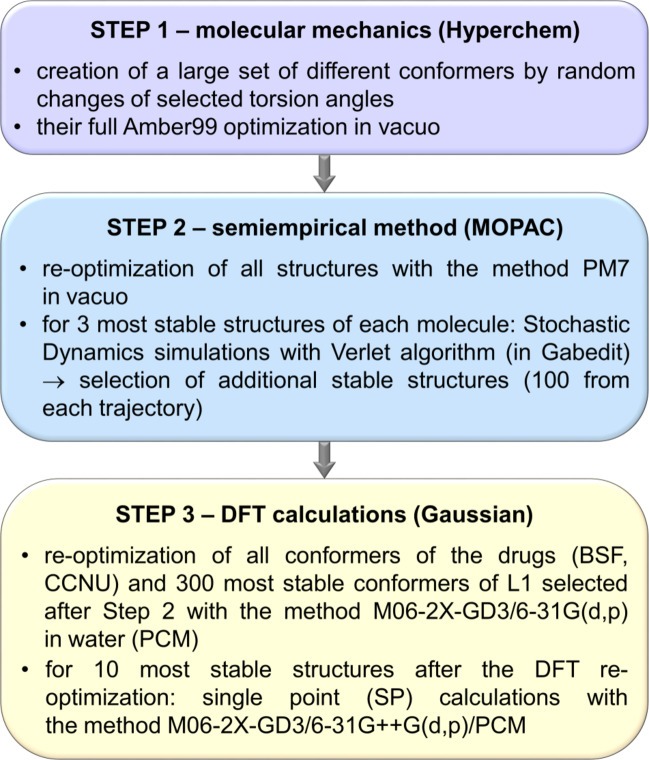
Flow chart of the procedure used in the search for the lowest energy conformers of the molecules L1, BSF and CCNU. The torsion angles varied are shown in Supplementary Fig. S17.

The procedure used for the isolated substrates is schematically depicted in Fig. 3 and described in more detail in Supplementary Procedure S1. Based on the results of the M06-2X-GD3/6-31G(d, p)/PCM calculations, the lowest energy conformer in water was selected for each molecule. These structures were used to create the initial models of the complexes L1:BSF and L1:CCNU.

#### Calculations for the complexes L1:BSF and L1:CCNU

**Fig. 4:**
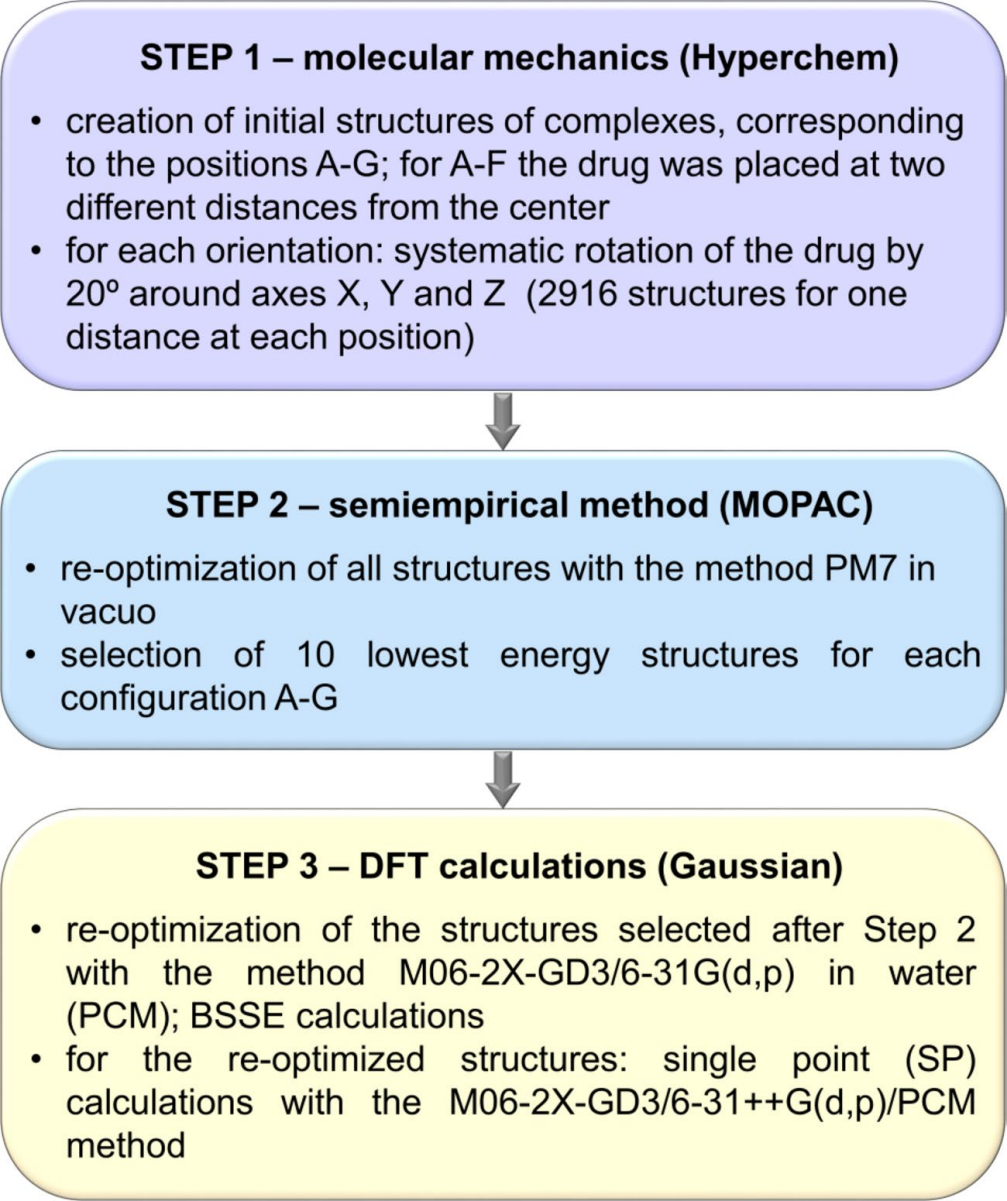
Flow chart of the procedure used in the search for the lowest energy configurations of the complexes L1:BSF and L1:CCNU. The configurations A-G are shown in Supplementary Fig. S18.

The method applied in investigations of the complexation ability of L1 towards BSF and CCNU is presented in Fig. 4 and described in more detail in Supplementary Procedure S2. Also in this case the “hierarchical” approach was used, but the general procedure was different, as for each complex seven different positions of the drug with respect to L1 were considered (Supplementary Fig. S18). The final results were obtained with the M06-2X-GD3/6-31G(d, p)/PCM method using the Gaussian 09 and 16 packages^44,53^. Additionally, for all optimized structures, the single point (SP) calculations were performed with the same DFT method and the larger basis set 6–31 + + G(d, p) that includes diffuse functions.

The complexation energies were calculated as: $\:{E}_{\text{c}\text{o}\text{m}\text{p}\text{l}}={E}_{\text{L}1:\text{d}\text{r}\text{u}\text{g}}^{\text{O}\text{P}\text{T}}-\left({E}_{\text{L}1}^{\text{O}\text{P}\text{T}}+{E}_{\text{d}\text{r}\text{u}\text{g}}^{\text{O}\text{P}\text{T}}\right)$, where $\:\:{E}_{\text{L}1:\text{d}\text{r}\text{u}\text{g}}^{\text{O}\text{P}\text{T}}$, $\:{E}_{\text{L}1}^{\text{O}\text{P}\text{T}}$ and $\:{\:E}_{\text{d}\text{r}\text{u}\text{g}}^{\text{O}\text{P}\text{T}}\:$ are the total energies of the optimized complex L1:drug and of the L1 and drug molecules. The M06-2X-GD3/6-31G(d, p) complexation energies were corrected by the basis set superposition error (BSSE) calculated using the counterpoise correction^54^.

The vibrational analysis was performed with the M06-2X-GD3/6-31G(d, p) method (PCM water) at 298.15 K and 1 atm of pressure. The Gibbs energies were additionally recalculated in the program GoodVibes v2.0.3^55^ to include the Grimme corrections for low vibrational frequencies (wave numbers < 100 cm^− 1^)^56^ as well as to account for the change of volume available to each molecule in solution when compared to gas phase and for the concentration change to 1 mol l^− 1^^57^. The corrected Gibbs energies are denoted below as *G*_corr_. The SP 6–31 + + G(d, p) complexation Gibbs energies were obtained by adding the thermodynamic correction obtained with the 6-31G(d, p) (without BSSE) to the SP energy.

The ^1^H NMR calculations were performed at the M06-2X/6–31 + + G(d, p)//M06-2X-GD3/6-31G(d, p) theory level using the Gauge-Independent Atomic Orbital (GIAO) approach^58^. The structures of all compounds obtained from their optimization in water were first re-optimized in DMSO. The final chemical shifts *δ* for protons in DMSO (relative to TMS) were calculated using the procedure and formula *δ*=(I−σ)/(−S) proposed by Tantillo^59^, where σ are the isotropic values obtained from DFT calculations, and I and S are the scaling factors. Their values obtained for protons with the method M06-2X/6–31 + + G(d, p)//M06-2X-GD3/6-31G(d, p) in DMSO (PCM) are: I = 32.0355 and S=-1.1608.

To explore the solvent effect on the final structure of L1, additional calculations were performed for a cluster of 20 water molecules and three selected conformers of L1: the most stable one found with the M06-2X-GD3/6-31G(d, p)/PCM method and for two “open” structures taken from the inclusion complexes G of L1:BSF and L1:CCNU. Similar tests with models including explicitly 20 H_2_O were done for the most stable non-inclusion complexes L1:BSF and L1:CCNU as well as for their inclusion forms. The models of these systems were selected after the molecular mechanics and semiempirical Molecular Dynamics simulations, as described in detail in Supplementary Procedure S3. All selected models containing 20 H_2_O were then fully optimized with the M06-2X-GD3/6-31G(d, p)/PCM (water) method.

### Cytotoxicity measurement

Breast cancer cells MCF-7, colon cancer cells HT-29, normal breast epithelial cells MCF-10 A and normal colon fibroblasts CCD-18Co were used as a cell model for testing the effect of compounds on the viability of cells. The cells were seeded in Dulbecco’s Modified Eagle Medium (DMEM) medium in 96-well plates and incubated for 24 h in a 5% CO_2_ atmosphere at 37 °C. MCF-10 A cells were grown in a medium supplemented with 0.4% bovine pituitary extract (BPE), 3 ng ml^− 1^ hEGF, 5 µg ml^− 1^ insulin, 0.5 µg ml^− 1^ hydrocortisone and 100ng mL^− 1^ cholera toxin. After that, cells were treated with different concentrations: 2.5, 10, 25, 50, 100, and 200 µM, of compounds for 72 h. Busulfan and lomustine were freshly dissolved in DMSO immediately before cell treatment. The solution of other compounds (lyophilized) were prepared in 30% DMSO. Then, a series of dilutions were prepared. The final concentrations of drugs, cryptand and complexes were in range 2.5–200 µM. The final concentration of DMSO diluted in culture medium was 0.2% for all experiments. The controls (without drugs) were treated with 0.2% DMSO. In this concentration DMSO was not cytotoxic for cells which was confirmed by preliminary experiments. The viability of cells treated with the tested compounds was assessed by measuring the ability of live cells to metabolize 3-(4,5-dimethylthiazolo-2-yl)-2,5-diphenyl tetrazolium bromide (MTT) to formazan, using a standard protocol. Briefly, after incubation with compounds, the medium of each well was discarded, cells were washed with PBS, and then 100 µL of fresh media with 10 µL MTT solution (5 mg mL^− 1^ in PBS) was added to each well. After 4 h incubation at 37 °C, the produced formazan was solubilized by the addition of DMSO, and the absorbance of each well was determined at 570 nm using an ELISA reader. The results were expressed as the mean of at least four replicates as a percentage of control (taken as 100% - cells).

## Results and discussion

### Experimental evidence of formation of complexes L1:BSF and L1:CCNU

The cryptand L1 proved to form complexes with both BSF and CCNU, which was confirmed by a comparative analysis of the ^1^H NMR spectra of pure components and their complexes. The signal shifts Δδ indicating complexation are presented in Table 1; Figs. 5 and 6, while the full spectra for L1, BSF, CCNU and their complexes as well as the ^1^H NMR 2D COSY spectrum for L1 are given in Supplementary Figs S6-S16. Atom numbers used in the discussion below are shown in Fig. 1.

**Fig. 5:**
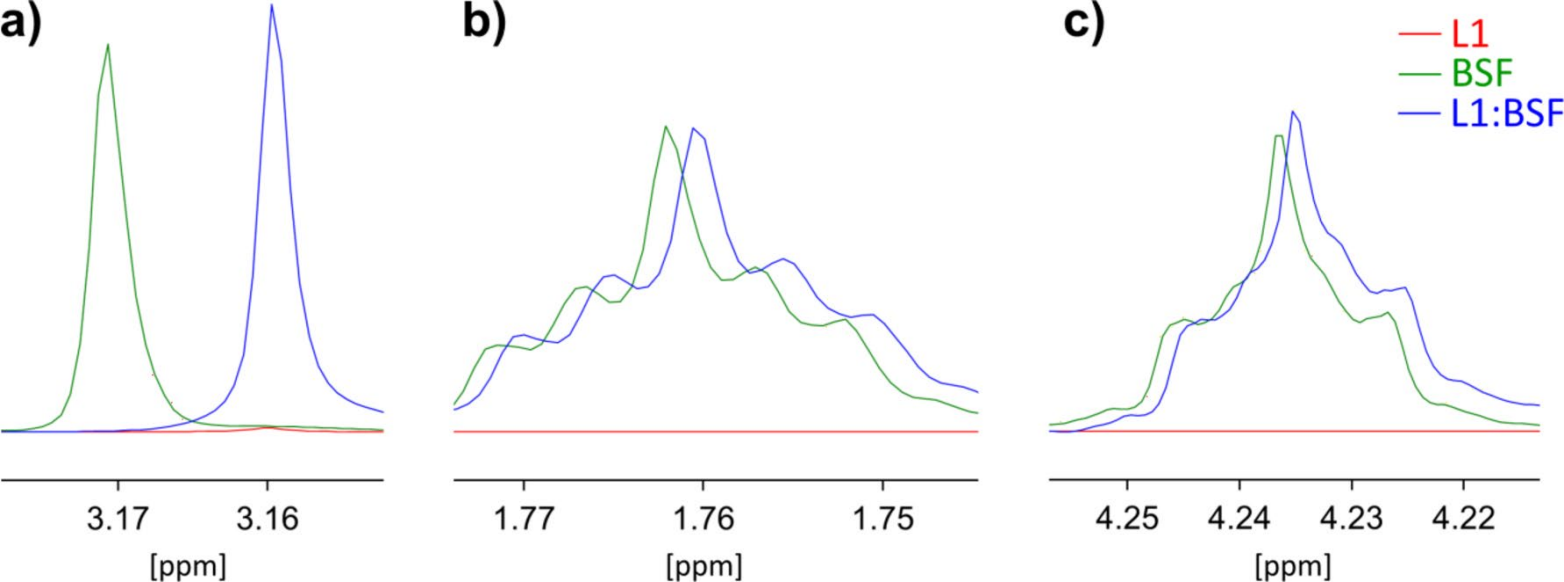
^1^H NMR chemical shifts positions in DMSO-d_6_ for protons in the pure and complexed BSF: (**a**) H-3 (CH_3_), (**b**) H-2 (CH_2_), (**c**) H-1 (OCH_2_).

For both BSF and L1 in the complex L1:BSF, the changes Δδ are rather small, but still visible. In the signals of BSF, the largest shift of proton signals is observed for the methyl group (Fig. 5a), but some displacements appear also for the remaining protons (Fig. 5b,c). Assuming that the CH_3_ group is adjacent to the center forming a hydrogen bond with the hydroxyl group of saccharides, this will obviously result in a shift in the position of the protons BSF_H-3 in the complex L1:BSF. The spectrum of L1 is very complex and difficult to interpret, because in the range of 4 − 3 ppm the signals from hydroxyl protons overlap with these from azacrown ether protons. Therefore, only a few signals could be assigned to specific protons. Among them, the most prominent Δδ is found for L1_OH-2 (Table 1), suggesting that this proton is somehow involved into formation of the complex with BSF. In the full spectrum (Supplementary Fig. S13), displacements of other L1 chemical shifts, which could not be identified, can be also seen, for example in the range of 3.7–3.2 ppm (Supplementary Fig. S14).

**Fig. 6:**
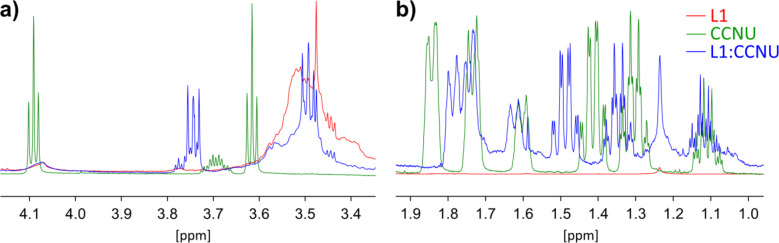
^1^H NMR chemical shifts positions in DMSO-d_6_ for protons in the pure and complexed CCNU: (**a**) H-7 and H-8, (**b**) H-2 to H-6 (cyclohexyl fragment).

In the case of L1:CCNU, an effect of the complex formation is much stronger manifested in the NMR spectra, especially when the chemical shifts for CCNU are compared. Significant displacements of signals are observed for the protons CCNU_H-8 and CCNU_H-7 from the methylene groups (Fig. 6a). They are close to oxygen atoms, which can form hydrogen bonds with hydroxyl groups from saccharide. Somewhat smaller, but also visible are the signal shifts for protons in the cyclohexane group (Fig. 6b). Also for protons in the pure host L1 and its complex with CCNU, clear difference Δδ is observed for L1_OH-2 (Table 1).

For all compounds considered in this study, an attempt was made to estimate and compare their solubility in water and the results are summarized in Table 2. As can be seen, the macrocycle L1 is very well soluble in water and, as a potential drug carrier, can significantly enhance the aqueous solubility of drugs. Indeed, the water solubility values for L1:BSF and L1:CCNU are about 46 and 240 times higher, respectively, compared to the solubility of pure drugs.

**Table 1:** The ^1^H NMR chemical shifts δ (the center of the signal for individual atoms) in DMSOd_6_ for selected protons in the pure drugs, cryptand L1 and in the complexes L1:BSF and L1:CCNU, and their change Δδ upon complexation.

Compounds	Proton no.	δ pure compound	δ complex	Δδ
BSF in L1:BSF	H-1	4.2365	4.2352	− 0.0013
	H-3	3.1707	3.1598	− 0.0109
	H-2	1.7620	1.7605	− 0.0015
L1 in L1:BSF	NH-1	7.4755	7.4791	0.0036
	NH-6	7.1075	7.1097	0.0022
	OH-2	5.7584	5.8224	0.0640
	OH-3	5.6772	5.6787	0.0015
	OH-4	4.5615	–	–
	H-1	4.5575	4.5572	-0.0003
CCNU in L1:CCNU	HN	8.5045 (d)	8.5220 (s)	0.0175
	H-8	4.0838	3.7443	− 0.3395
	H-1	3.6866	3.7501	0.0635
	H-7	3.6078	3.4932	− 0.1146
	H-2a, H-6a	1.8356	1.7884	− 0.0472
	H-3a, H-5a	1.7271	1.7447	0.0176
	H-4a	1.5948	1.6228	0.0280
	H-2b, H-6b	1.4064	1.4876	0.0812
	H-3b, H-5b	1.2957	1.3462	0.0505
	H-4b	1.1004	1.1170	0.0166
L1 in L1:CCNU	NH-1	7.4755	7.4754	− 0.0001
	NH-6	7.1075	7.1086	0.0011
	OH-2	5.7584	5.7457	− 0.0127
	OH-3	5.6772	–	–
	OH-4	4.5615	–	–
	H-1	4.5575	4.5572	− 0.0003

All values are in ppm.

**Table 2:** Solubility of compounds in water.

Compounds	Solubility [mg ml^− 1^]
L1	20.0
BSF	0.13
L1:BSF	6.0
CCNU	0.05
L1:CCNU	12.0

### DFT calculations for the isolated molecules and their complexes

#### Structural and energy parameters obtained with an implicit water model

**Fig. 7:**
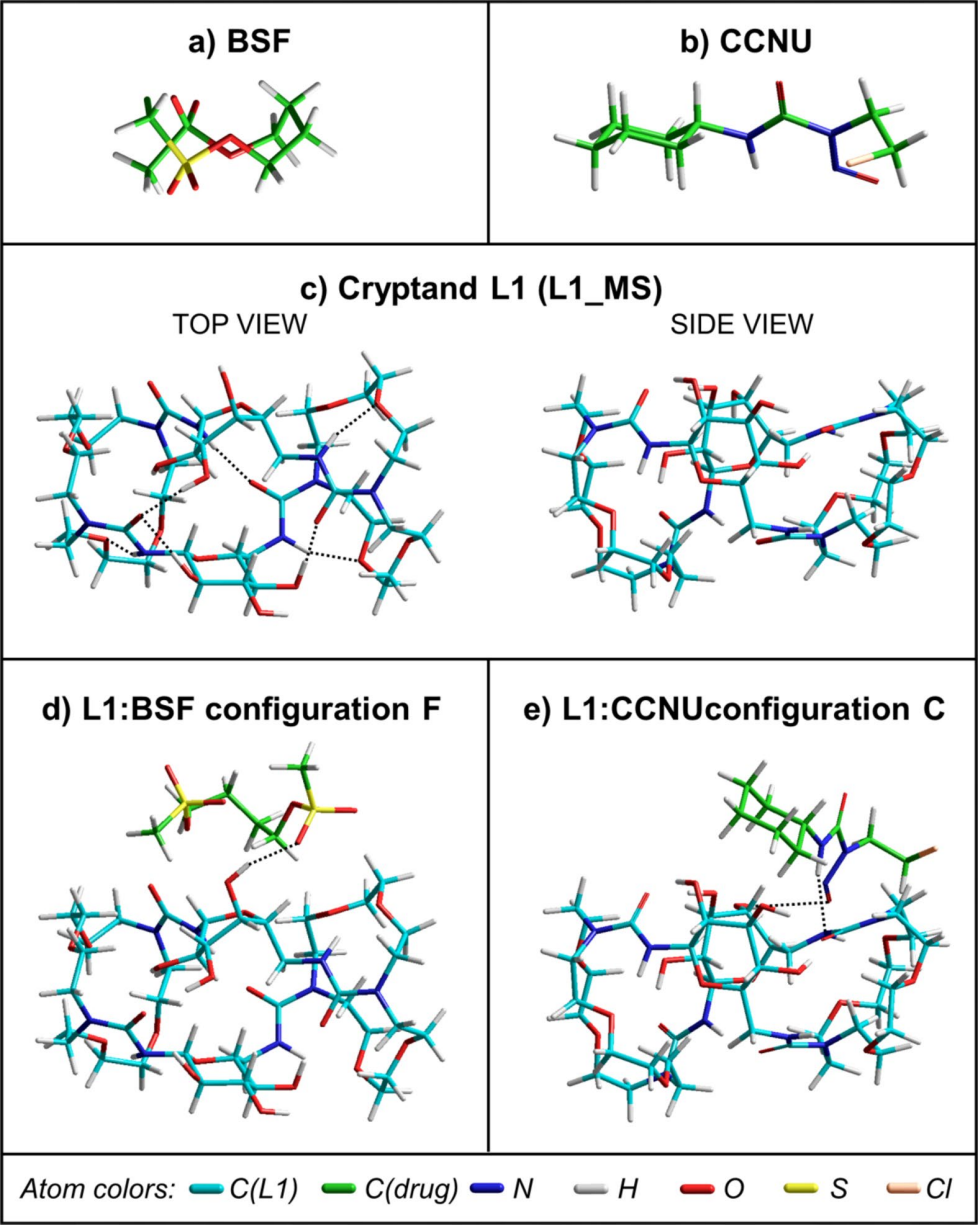
The lowest energy structures of the substrates (**a**-**c**) and the most stable configurations of the complexes L1:BSF (**d**) and L1:CCNU (**e**), obtained from the M06-2X-GD3/6-31G(d, p) optimizations in water (PCM). For the macrocycle L1 alone, both “top” and “side” views are shown. To distinguish the two molecules forming the complex, different colors were used for the carbon atoms in the drugs (green) and in L1 (cyan). The black dashed lines indicate the most prominent hydrogen bonds in L1 alone (**c**) as well as between L1 and the drugs in their complexes (**d**,**e**).

Quantum chemical calculations allowed us to obtain a more detailed insight into the structures and stability of the macrocycle L1, both drugs and their complexes in stoichiometry 1:1. The lowest energy structures of these compounds found in the present work with the M06-2X-GD3/6-31G(d, p) method in water (PCM) are presented in Fig. 7. The single point calculations performed for the complexes with the larger basis set 6–31 + + G(d, p) confirmed that these are the most stable structures (Supplementary Table S1).

The conformer of BSF shown in Fig. 7a differs from that found in the earlier work of Karthick et al.^28^ from the B3LYP-D/6-311 + + G(d, p) calculations in vacuo. The structure in Fig. 7a is less symmetric and the methanesulfonyl groups are placed closer to each other than in the conformer B presented in ref. 28. It should be emphasized, however, that in the present work a larger configurational space was explored (seven torsion angles were varied) and, moreover, the DFT calculations were performed in water. According to the M06-2X-GD3/6–31 + + G(d, p)/PCM results obtained for the ten lowest energy structures of BSF, the relative energies ΔE, calculated with respect to the energy of the most stable conformer, are rather small, within 1.1 kcal mol^− 1^.

Also the lowest energy conformer of CCNU shown in Fig. 7b differs from that obtained earlier by Cao et al.^29^ from their B3LYP/6-311 + + G(d, p) calculations performed in the gas phase. However, in ref. 30 only six different geometries of CCNU were tested and the structure shown in Fig. 7b was not included in this set. Similar to BSF, the relative energies ΔE of the first ten conformers are within a relatively narrow range of 1.63 kcal mol^− 1^.

Contrary to what one might expect from Fig. 1, the geometry of the most stable conformer of L1 (L1_MS; Fig. 7c) is not regular. Due to the formation of intramolecular hydrogen bonds, the structure is elongated and rather compact. For this molecule, the SP relative energies are larger, and for the 2nd conformer ΔE is of about 1 kcal mol^− 1^, while for the 10th it is 8.5 kcal mol^− 1^. However, all these conformers do not possess a typical cavity enabling the formation of an inclusion complex.

**Fig. 8:**
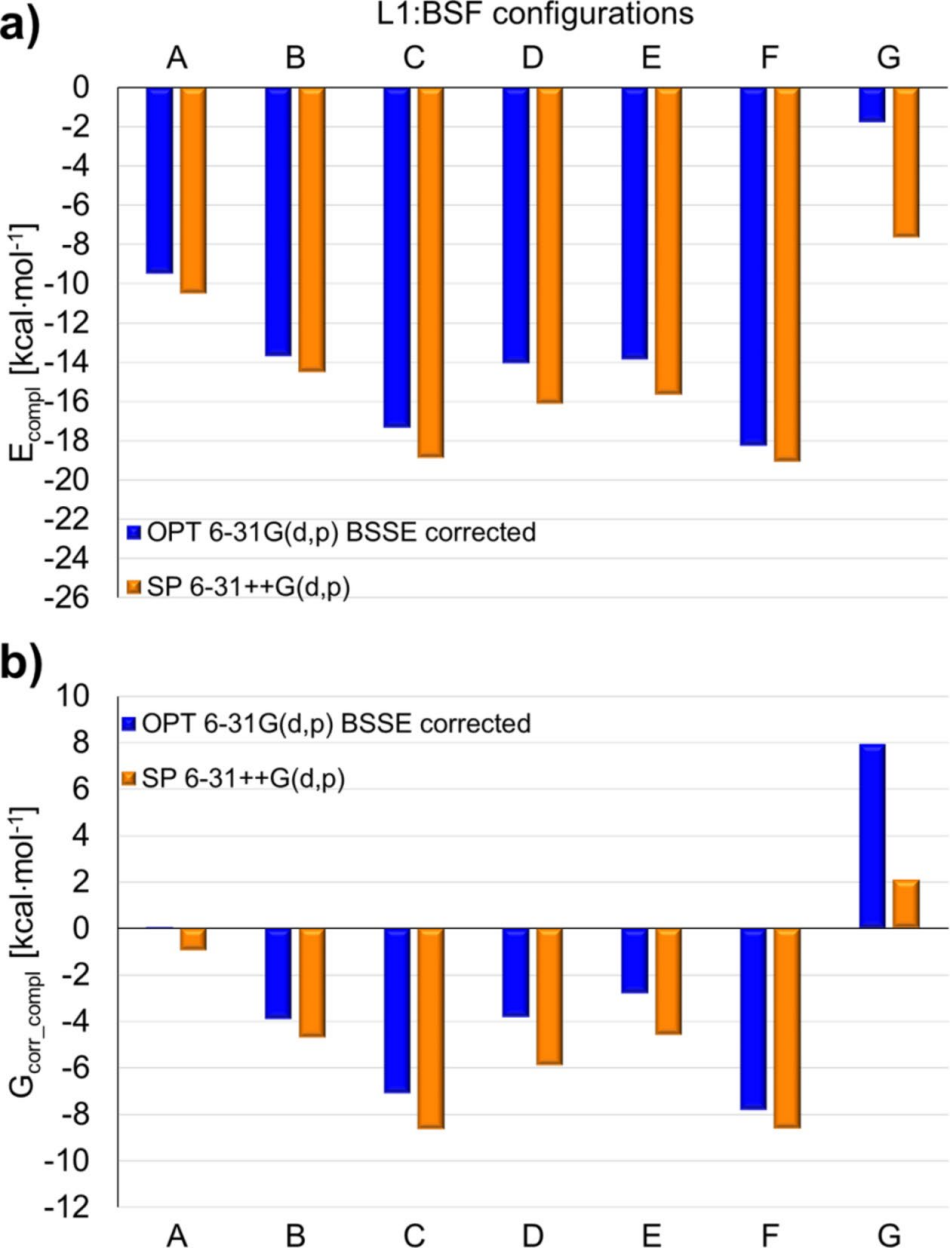
The complexation energies (**a**) and the corresponding Gibbs energies (**b**) for the complex L1:BSF in its seven different configurations A-G shown in Supplementary Fig. S19, obtained in water (PCM) from the M06-2X-GD3/6-31G(d, p) optimizations (OPT; include the BSSE corrections) and from the SP calculations performed with the M06-2X-GD3/6–31 + + G(d, p) method (SP; without BSSE). The numerical values are given in Supplementary Table S2.

**Fig. 9:**
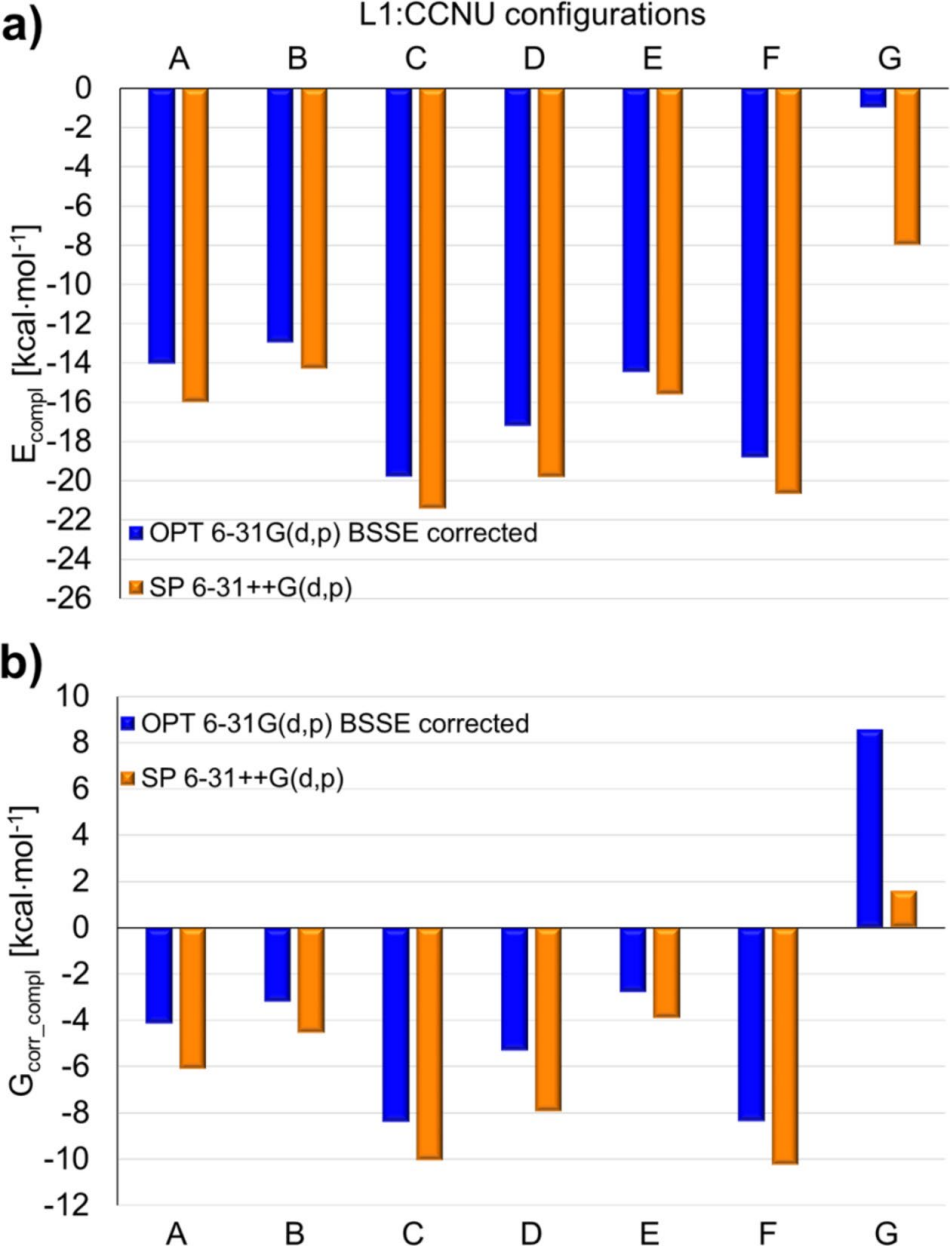
The complexation energies (**a**) and the corresponding Gibbs energies (**b**) for the complex L1:CCNU obtained from the DFT calculations in water (PCM) (Supplementary Fig. S20). The numerical values are given in Supplementary Table S2.

Indeed, the complexation energies E_compl_ obtained for different configurations of the L1:BSF (Fig. 8a) and L1:CCNU complexes (Fig. 9a) indicate that it is energetically more favorable to attach the drug externally to L1 (structures A-F in Supplementary Figs S19, S20) than to form an inclusion complex (structures G in Supplementary Figs S19, S20). For L1:BSF, the most stable is the complex F (Fig. 7d), followed by C and D. In the case of L1:CCNU, also these three configurations are favored, but the ordering is different, as the complex C (Fig. 7e) is more stabilized than F and D (Supplementary Table S2). For both L1:BSF and L1:CCNU, the absolute values of the SP 6–31 + + G(d, p) complexation energies are slightly larger, but this does not change the overall trend in the relative stability of the individual configurations.

In four L1:BSF complexes (C, E, F, G) and all L1:CCNU complexes, one or two hydrogen bonds can be observed between the drug and L1. Those occurring in the most stable complexes are marked in Fig. 7e, f. However, their presence is not the only factor influencing the stability of the complexes, as it also depends on other interactions, such as electrostatic and dispersion forces, as well as on the energy cost associated with molecular deformation. As can be seen in Supplementary Table S2, for both complexes the strongest drug-cryptand interactions occur in the inclusion complexes G, for which the interaction energies E_int_ are − 42.7 (L1:BSF) and − 36.4 kcal mol^− 1^ (L1:CCNU). These values are much more negative than for all other configurations. However, placing the drug inside the cryptand causes a significant distortion of L1, which is reflected by very large values of its deformation energy E_def_L1_ (30.6 in L1:BSF and 25.8 kcal mol^− 1^ in L1:CCNU). As a result, the complexation energies for the inclusion complexes are much smaller than for the other configurations. The deformation energies for both drugs (E_def_drug_) are relatively small and do not have such a large impact on the stability of the complexes.

The Gibbs energies calculated for L1:BSF and L1:CCNU indicate that, except for the inclusion complex G, all other configurations should be formed spontaneously in water at the room temperature (Figs. 8b and 9b). For both complexes, the most negative values correspond to the structures F and C. This is due to the fact that the magnitude of the entropy term TdS is similar for all the configurations studied: for L1:BSF it has values ranging from − 8.1 to -9.3 kcal mol^− 1^, while for L1:CCNU – from − 8.2 to -10.2 kcal mol^− 1^.

#### Computed 1H NMR chemical shifts in DMSO

For the most stable structures of the isolated substrates and selected complexes obtained in water and re-optimized in DMSO, ^1^H NMR chemical shifts δ in DMSO and their changes Δδ were calculated and compared to the experimental data. It should be mentioned, that both structures and complexation energies obtained after re-optimization in DMSO are very similar to those in water. The root-mean square deviation (RMSD) of atomic positions for the structures L1:BSF F and L1:CCNU C re-optimized in DMSO, calculated relative to their water-optimized structures, are 0.0024 and 0.004 Å, respectively. The complexation energies obtained for these two complexes in DMSO (without BSSE) are lower than these in water (also without BSSE) by only 0.053 and 0.066 kcal mol^− 1^. The averaged calculated ^1^H NMR chemical shifts (Supplementary Table S3) differ significantly from the measured values. This is not surprising, as the DFT results are obtained for a single structure of each compound, while in a real solution various configurations probably co-exist. It is worth noting the large difference in the δ values for L1_OH-2 for the F and G configurations of L1:BSF and for C and G of L1:CCNU. Only in the inclusion complexes G δ for this proton is around 5.5 ppm, which is close to the experimental values for L1 and both complexes. However, in the G complexes, the proton L1_OH-2 forms the hydrogen bond with oxygen belonging to the closest urea bridge. This may suggest that such arrangement is also present in L1 alone, therefore in the real DMSO-d_6_ solution its structure differs from the most stable L1_MS. However, unlike deduced from the measured ^1^H NMR spectra, L1_OH-2 does not appear to be directly involved into interaction with the drug in the complexes. On the other hand, the implicit solvent model does not describe properly specific interactions of DMSO with the OH and NH groups, which are expected to affect the δ values.

This explains also discrepancies that are seen in the changes Δδ upon complexation, which were calculated with respect to δ of L1_MS. Although both calculated and measured results indicate the formation of the L1:drug complexes, the Δδ values are different. For example, the experimental Δδ values for BSF in L1:BSF are negative and very small, indicating rather weak interaction between L1 and BSF. The calculated Δδ are quite large and, except for BSF_H-2 in the configuration F, positive. The ^1^H NMR chemical shifts obtained from the DFT calculations for all atoms in the molecules studied are given in Supplementary Tables S4-S6.

#### The influence of intermolecular interactions with water molecules on the stability of L1, L1:BSF and L1:CCNU

The discussion above is based on the results obtained using the implicit solvent model PCM, which does not take into account specific interactions, such as hydrogen bonds between the macrocycle L1 and water molecules. As mentioned above, all low energy structures of L1 found with the PCM model are twisted and therefore preferentially form non-inclusion complexes. Some additional tests were performed for two “open” structures, L1_O1 and L1_O2, extracted from the inclusion complexes G of L1:BSF and L1:CCNU (Supplementary Fig. S22). To check if there are nearby local minima corresponding to similar “open” geometry, they were optimized using the PCM water model, but as a result they adopted a compact structure.

However, this could be an artefact caused by the implicit solvent model, therefore additional calculations were performed for L1_MS, L1_O1 and L1_O2 with 20 water molecules included explicitly. Similar calculations were performed for the most stable complexes L1:BSF and L1:CCNU and their inclusion configurations G. The resulting structures and energies are presented in Supplementary Figs S22, S23 and Tables S7, S8. The interactions with 20 waters cause some deformation of all molecules, which is clearly seen in the values E_def_vs_PCM_ in Supplementary Table S8. The most important result is that, in the presence of 20 H_2_O, the structures of L1_O1 and L1_O2 after full optimization retain their geometries close to the initial ones, so they do not collapse. L1_O1 is more “open” and three water molecules were found to be present inside its cavity, only one in L1_O2 and none in L1_MS (Supplementary Fig. S22). The total energy of L1_O1:20H_2_O is higher than that of L1_MS:20H_2_O by only 1 kcal mol^− 1^. So small energy difference results from stronger interactions of L1_O1 with 20 H_2_O (E_int_ of -157 vs. -124 kcal mol^− 1^ for L1_MS, Supplementary Table S8), which compensate for the deformation of L1 in L1_O1:20H_2_O (E_def_rel_=33 kcal mol^− 1^) with respect to its structure in L1_MS:20H_2_O. A similar stabilizing effect of interactions with water molecules is also visible for the complexes. The relative energy differences ΔE_rel_ between the complexes G and F of L1:BSF_20H_2_O and G and C of L1:CCNU_20H_2_O are 8.8 and 7 kcal mol^− 1^, respectively, and they are lower than those obtained in water described only by the implicit PCM solvent model (12.6 and 17.1 kcal mol^− 1^, respectively).

### Evaluation of cytotoxicity

**Fig. 10:**
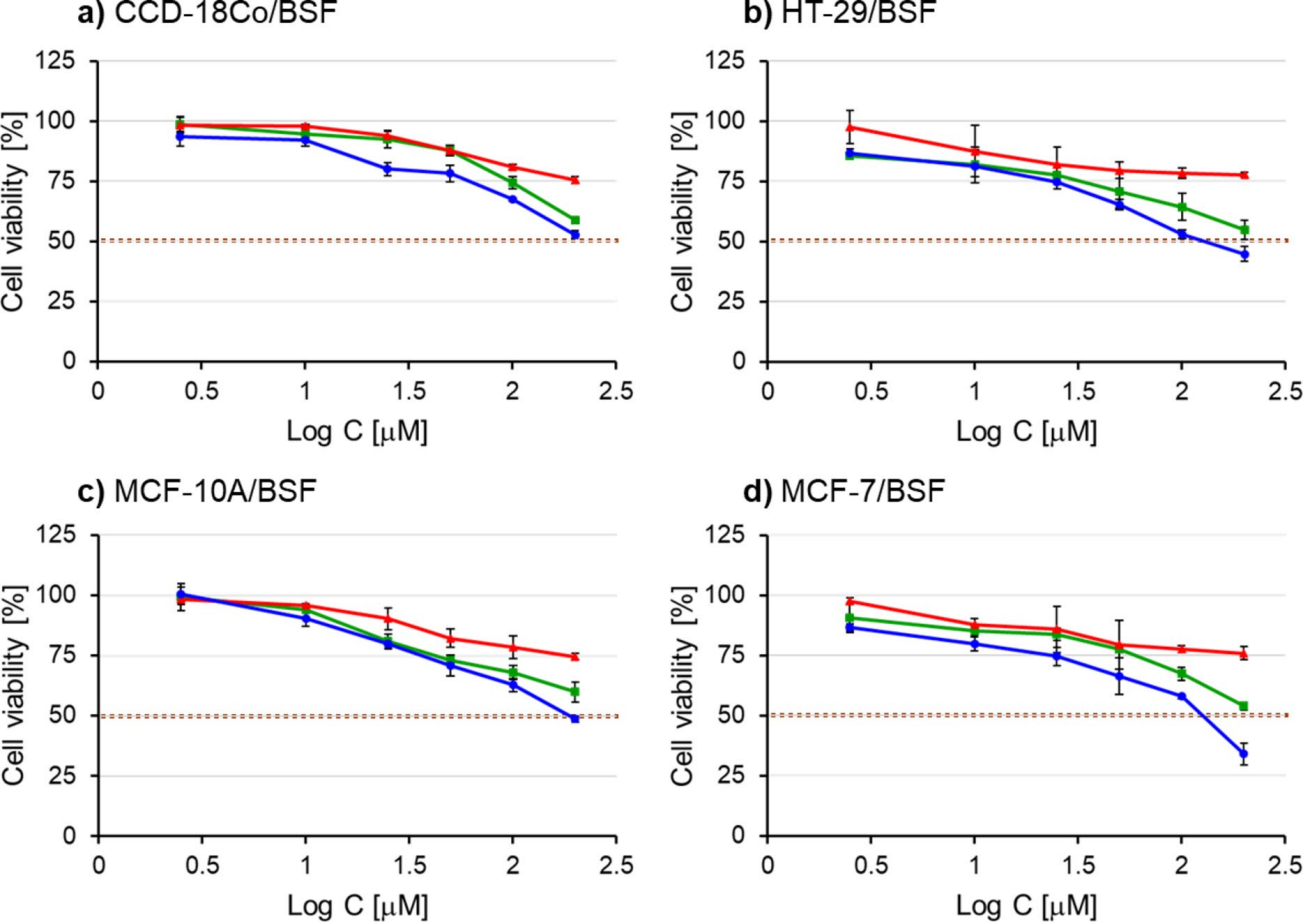
An effect of cryptand L1, pure BSF and L1:BSF complex on the viability of normal and cancer cells. Human colon normal CCD-18Co (**a**) and cancer HT-29 (**b**) cells as well as breast normal and cancer cells, i.e. MCF-10 A and MCF-7 (**c** and **d**, respectively), were treated for 72 h with different concentrations of L1 (red line), BSF (green line) and L1:BSF (blue line).

**Fig. 11:**
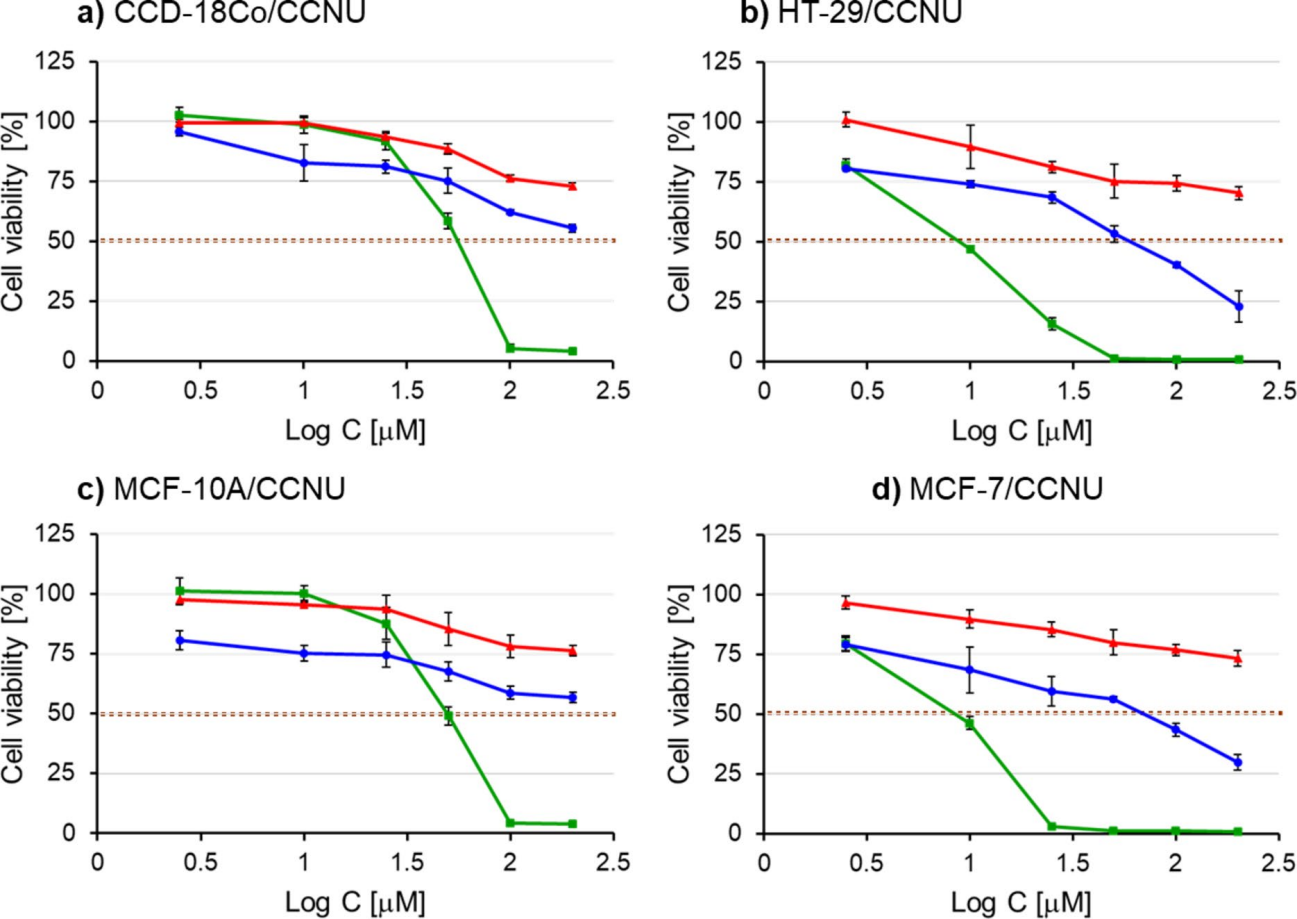
An effect of cryptand L1, CCNU and L1:CCNU complex on the viability of normal and cancer cells. Human colon normal CCD-18Co (**a**) and cancer HT-29 (**b**) cells as well as breast normal and cancer cells, i.e. MCF-10 A and MCF-7 (**c** and **d**, respectively) were treated for 72 h with different concentrations of L1 (red line), CCNU (green line) and L1:CCNU (blue line).

The suitability of cryptand L1 as a potential drug carrier was assessed by examining its effect on the cytotoxicity of BSF and CCNU. Cell viability was determined through the MTT assay in normal and cancer breast cells (MCF-10 A and MCF-7, respectively) as well as normal and cancer colon cells (CCD-18Co and HT-29) treated with the cryptand L1 alone, pure drugs BSF or CCNU, and the cryptand-drug complexes (Figs. 10 and 11). The cytotoxic effect was expressed as IC_50_, which is the drug concentration causing a 50% growth inhibition. The IC_50_ values are presented in Table 3 as mean ± standard deviation. The mean was calculated based on four replicates. The cryptand alone showed low cytotoxicity and the IC_50_ value had not been reached in the used concentration even after long incubation of cells with the compound, i.e. 72 h. Moreover, there was no significant difference in the sensitivity of cells for L1.

The two tested drugs had different effects on cell viability. As in the work of Kokotos et al.^20^, the cytotoxicity of BSF towards all cells tested was generally very low. For pure BSF, the IC_50_ value was not achieved in the concentration range used. However, the L1:BSF complex showed cytotoxic activity. Our results suggest that L1:BSF is more cytotoxic for cancer cells than normal cells, however, possible selectivity towards tumorigenic cells needs to be proved in future studies.

Unlike BSF, CCNU was highly cytotoxic to all cells. The IC_50_ values for nontumoral CCD-18Co and MCF-10 A cells treated with CCNU alone were 56.2 µM and 48.9µM, respectively. Cancer cells were more sensitive and IC_50_ was obtained with a much lower concentration of the drug (8.91 and 8.41 µM for HT-29 and MCF-7, respectively). The complex L1:CCNU exhibits generally lower cytotoxicity and, in used concentration range, IC_50_ was obtained only for cancer cells. This effect may possibly be due to a reduction in drug accumulation in cells.

**Table 3:** Cytotoxicity assay – IC_50_ values (mean ± standard deviation; µM).

Cell line	BSF	L1:BSF	CCNU	L1:CCNU
CCD-18Co	–	–	56.2 ± 7.8	–
HT-29	–	112.2 ± 9.4	8.91 ± 1.5	63.09 ± 2.0
MCF-10 A	–	188.36 ± 10.7	48.9 ± 5.4	–
MCF-7	–	63.02 ± 3.1	8.41 ± 1.6	66.83 ± 9.3

CCD-18Co and HT-29 denote normal and cancer human colon cells, respectively, while MCF-10 A and MCF-7 – normal and cancer breast cells.

## Summary and conclusions

In this work the cryptand containing two glucose units and two diazacrown ethers was tested as a potential drug carrier. The cryptand L1 is very well soluble in water, which causes the L1:BSF and L1:CCNU complexes to have much better solubility than pure drugs. According to the DFT results obtained with the implicit model of water (PCM), the most stable structure of L1 is twisted due to the presence of intramolecular hydrogen bonds between the sugar groups. Since there is no cavity in it where the drug could be placed, the formation of non-inclusion complexes is energetically favored. Their BSSE corrected complexation energies vary from − 18 to -10 kcal mol^− 1^ for L1:BSF and from − 20 to -13 kcal mol^− 1^ for L1:CCNU. For all of them the complexation process is predicted to be exothermic and spontaneous (negative ΔG). In the case of inclusion complexes, the calculated complexation energies are much smaller and ΔG is positive. However, further studies carried out with 20 H_2_O showed that different conformers of L1 can co-exist in solution, including the “open” structures with a cavity suitable for the formation of inclusion complexes. In the presence of 20 H_2_O, the total energy of such complexes is higher by only a few kilocalories per mole than the energy of the most stable non-inclusion complexes. This suggests that both inclusion and non-inclusion complexes can be present in a real aqueous solution. The results of cytotoxicity measurements showed that the cytotoxic activity of pure BSF against colon and breast cancer cells is very low. However, the L1:BSF complex showed good cytotoxic activity against cancer cells, especially the breast MCF-7 cells. Completely different results were obtained for CCNU and the L1:CCNU complex. Pure CCNU exerts a strong cytotoxic effect against all used types of cells. Interestingly, the complexation of CCNU by L1 lowered its cytotoxicity. All these results suggest that the cryptand L1 can be considered a potential drug carrier. However, further studies with various drugs are necessary to confirm the effectiveness of cryptand.

## Electronic supplementary material

Below is the link to the electronic supplementary material.

Supplementary Material 1

## Data Availability

The authors declare that the data supporting the findings of this study are available within the paper and its Supplementary Information files. Should any raw data files be needed in another format they are available from the corresponding author upon reasonable request.
