# Supplementary material for: Experimental and computational studies on possibility of using glucose diazacrown cryptand as a carrier for anticancer drugs busulfan and lomustine
Source: Sci Rep. 2024 Nov 18;14:28443. doi: 10.1038/s41598-024-80029-6 (PMC11574061; doi:10.1038/s41598-024-80029-6)
Supplement: Supplementary file 1 — Supplementary Material 1 [file 41598_2024_80029_MOESM1_ESM.pdf]

Experimental and computational studies on possibility of using glucose diazacrown cryptand as a carrier for anticancer drugs busulfan and lomustine

Anna Ignaczak,[a]* Marta Hoelm,[a] Stanisław Porwański,[b] Paweł Jóźwiak,[c] Anna Krześlak[c]

[a] *Theoretical and Structural Chemistry Group, Department of Physical Chemistry, Faculty of Chemistry, University of Lodz, 163/165 Pomorska St., 90-236 Lodz, Poland*

[b] *Department of Organic and Applied Chemistry, Faculty of Chemistry, University of Lodz, 12 Tamka St., 91-403 Lodz, Poland*

[c] *Department of Cytobiochemistry, Faculty of Biology and Environmental Protection, University of Lodz, 141/143 Pomorska St., 90-236 Lodz, Poland*

* Email: anna.ignaczak@chemia.uni.lodz.pl

Electronic Supporting Information

Table of Contents:

1. Experimental part S3

1.1. Synthesis details S3

1.1.1. General procedure of Staudinger–aza-Wittig (SAW) reaction S3

1.1.2. General of Zemplen procedure S3

1.1.3. Reaction with tosyl chloride general procedure S3

1.1.4. Reaction with sodium azide general procedure S3

1.1.5. Characterization of compounds by ^{1}H NMR measurement S3

1.2. Compounds identification S4

1.2.1. Intermediates in the synthesis of L1 (scheme in Figure 2) S4

1.2.2. NMR spectra for the cryptand L1, busulfan and lomustine S7

1.2.3. Superimposed NMR spectra of L1, drugs and complexes S11

2. Theoretical part S13

2.1. Details of calculation methods – procedures S1-S3 ....S13

2.2. Structures and energies of complexes in water (PCM) S17

2.3. Computed ^{1}H NMR chemical shifts S21

2.4. Structures and energies of L1, L1:BSF and L1:CCNU with 20 H_2O S26

2.5. Cartesian coordinates for the most stable structures S30

References S34

1. Experimental part

1.1. Synthesis details

1.1.1. General procedure of Staudinger–aza-Wittig (SAW) reaction

A solution (1mmol) of derivative D-glucose azide and triphenylphosphine (3mmol) in anhydrous toluene (20 ml) was stirred for 1h at r.t., then (0.5mmol) crown ether was added to the mixture, which was stired for 24 h under CO_2 bubbling. The reaction progress was controlled by thin-layer chromatography using plates chromatography plates (Merck TLC Silicagel60 F254). The plates were developed with sulfuric acid (VI) in methanol at high temperature. After evaporation of the toluene the residue was chromatographed on silicagel column (eluent AcOEt/MeOH).

1.1.2. General of Zemplen procedure

In a solution of MeOH/MeONa (50ml) the compound peracetyle (0.5mmol) was added. The resulting mixture was stirred at room temperature for 24 h and was filtered through a short column of resin Dovex 50WX8 hydrogen form. The filtrate was evaporated and the product was obtained as a white powder with quantitative yield and used without purification.

1.1.3. Reaction with tosyl chloride general procedure

The appropriate compound with free hydroxyl groups (1mmol) was dissolved in pyridine (10ml). Then the *p*-toluenesulfonyle chloride (1.5 mmol per one hydroxyl group) was added to the mixture which was stirred for 2 days a r.t. After this time, 6mmol of acetic anhydride was added to the reaction medium. After evaporation of the pyridine and acetic anhydride, the residue was chromatographed on a silica gel column (AcOEt/Hexane).

1.1.4. Reaction with sodium azide general procedure

The appropriate compound (1mmol) was dissolved in DMF (5ml). Then the sodium azide (5mmol) was added and the resulting mixture was stirred at 80°C for 12h. After cooling, ethyl acetate (50ml) was added and the mixture was washed with water (2x50ml). The organic phase was dried and after evaporation of the solvent was chromatographed on a silica gel column (AcOEt/Hexane).

1.1.5. Characterization of compounds by ^{1}H NMR measurement

Peracetylated sugar derivatives were purified by column or flash chromatography using methanol/ethyl acetate as eluents. Ligands with free hydroxyl groups after the Zemplen method were filtered through a layer of resin (Dovex 50WX8 hydrogen form) and silica gel and used directly. All samples for proton spectra were weighed at 0.01 mmol and dissolved in 1 ml of deuterated solvent. All spectra were taken in DMSO-d_6 of the same batch purchased from Merck and performed on the same Bruker Avance III (600MHz) instrument. The spectra were interpreted using the TopSpin 4.0.9 program.

1.2. Compounds identification

1.2.1. Intermediates in the synthesis of L1 (scheme in Figure 2)

1,10-*N,N'*-Bis-(1,2,3,4-tetra-*O*-acetyl-6-ureido-6-deoxy-*β*-D-glucosyl)-4,7,13,16-tetraoxa-1,10-diazacyclooctadecane (**5**) (procedure 1.1.1)
NMR ^{1}H (600MHz, $CDCl_3$); 6.36(tp, 2H, 2NH, urea); 5.20(t, 2H, 2H-3, J=9.5); 4.98(t, 2H, 2H, 2H-4, J=9.5); 4.95(t, 2H, 2H-2, J=9.2); 4.66(d, 2H, 2H-1, J=8.9); 3.75-3.46(m, 28H, 2H-6a, 2H-5, 24Hcrown); 4.43(ddd, 2H, 2H-6b, J=14.5, 1.0, 6.1) 2.08-2.00(3s, 18H,6CH_3, Ac); yield: 53%.

1,10-N,N'-Bis-(2,3,4-tri-O-acetyl-*β*-D-azide-6-ureido-6-deoxy-glucosyl)-4,7,13,16-tetraoxa-1,10-diazacyclooctadecane (**7**) (procedure 1.1.4)
IR(Nead, cm^{-1}): 2926, 2870, **2117**, 1756, 1755, 1668, 1640, 1239, 1215, 1047, 1062.
NMR ^{1}H (600MHz, $CDCl_3$); 6.29(t, 2H, 2NH, urea, $J_{NH,6}$=5.9); 5.22(t, 2H, 2H-3, J=9.9); 4.99(t, 2H, 2H, 2H-4, J=9.9); 4.97(t, 2H, 2H-2, J=9.5); 4.67(d, 2H, 2H-1, J=9.5); 3.75-3.46(m, 28H, 2H-6a, 2H-5, 24Hcrown); 3.42(ddd, 2H, 2H-6b, J=14.2, 2.0, 5.9) 2.09-2.01(3s, 18H,6CH_3, Ac); yield: 31%.

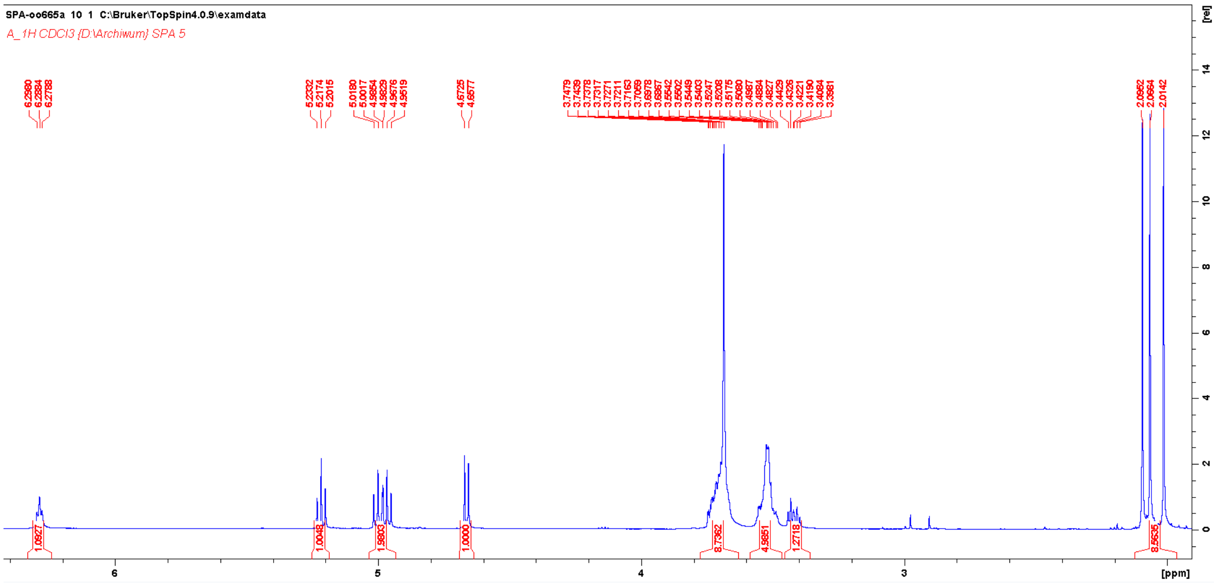

Figure S1. ^{1}H NMR spectrum for derivative **7** in $CDCl_3$.

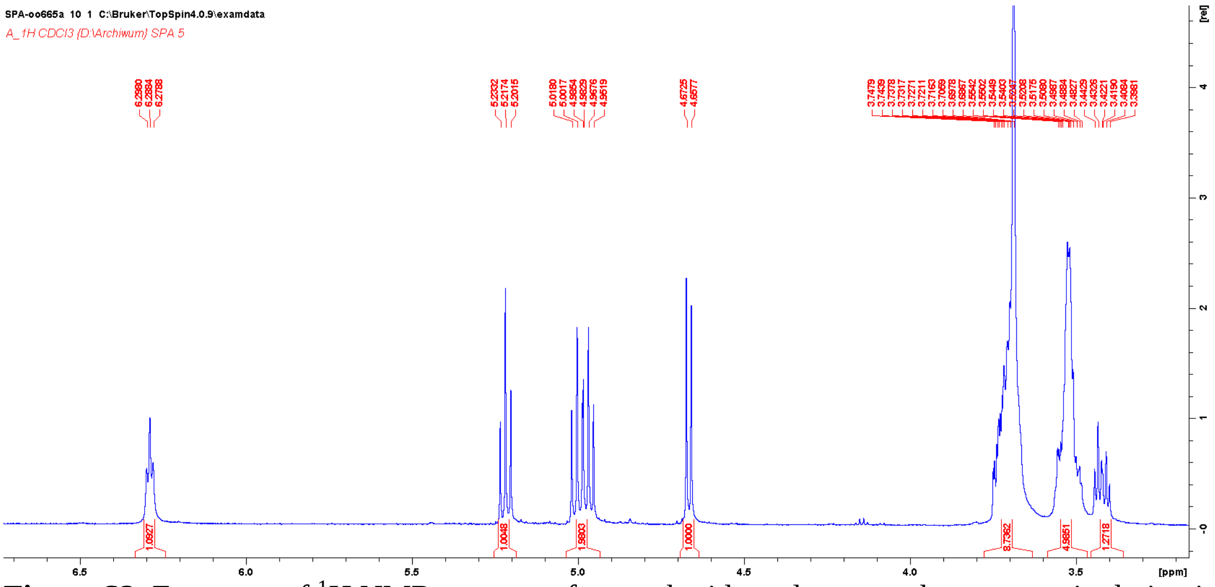

Figure S2. Fragment of ^{1}H NMR spectrum for saccharide and crown ether protons in derivative **7** in $CDCl_3$.

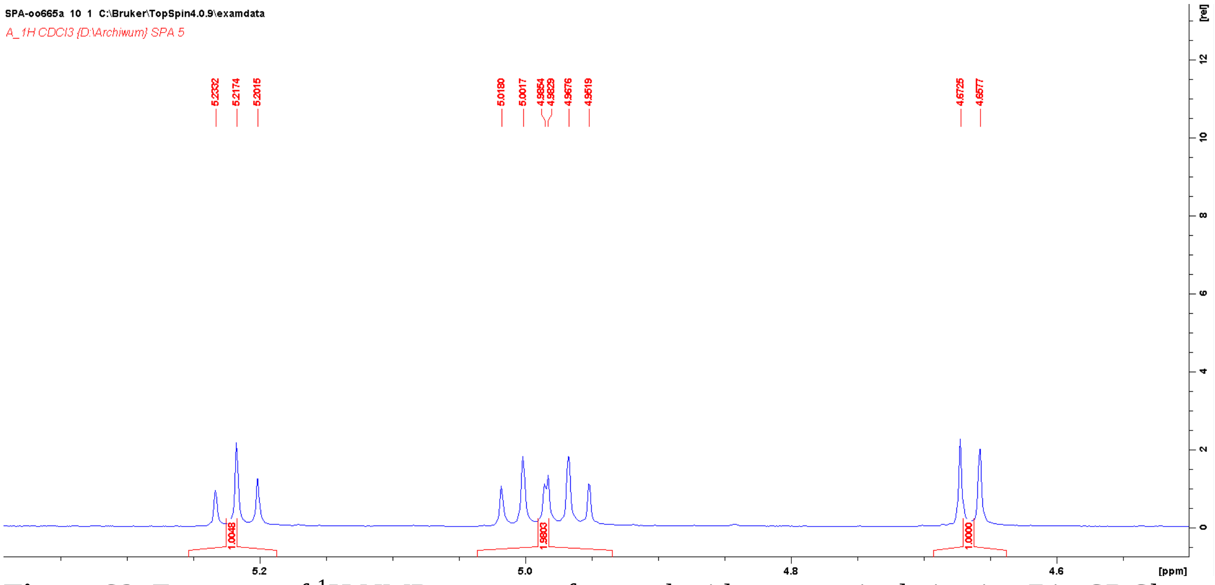

Figure S3. Fragment of ^{1}H NMR spectrum for saccharide protons in derivative **7** in $CDCl_3$.

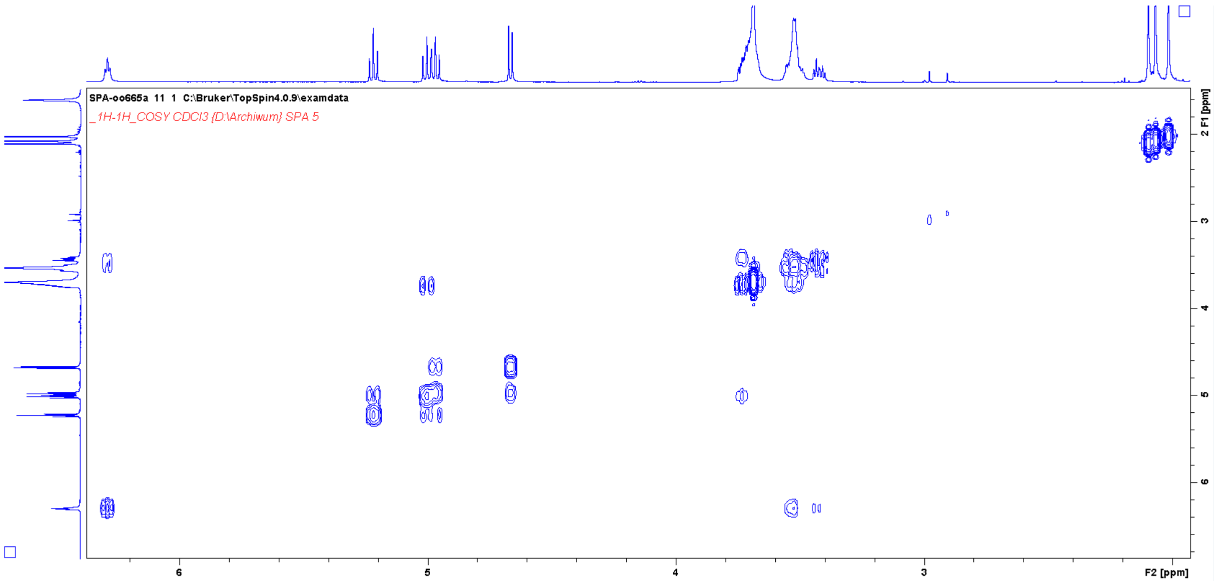

Figure S4. ^{1}H NMR 2D COSY spectrum for derivative **7** in $CDCl_3$.

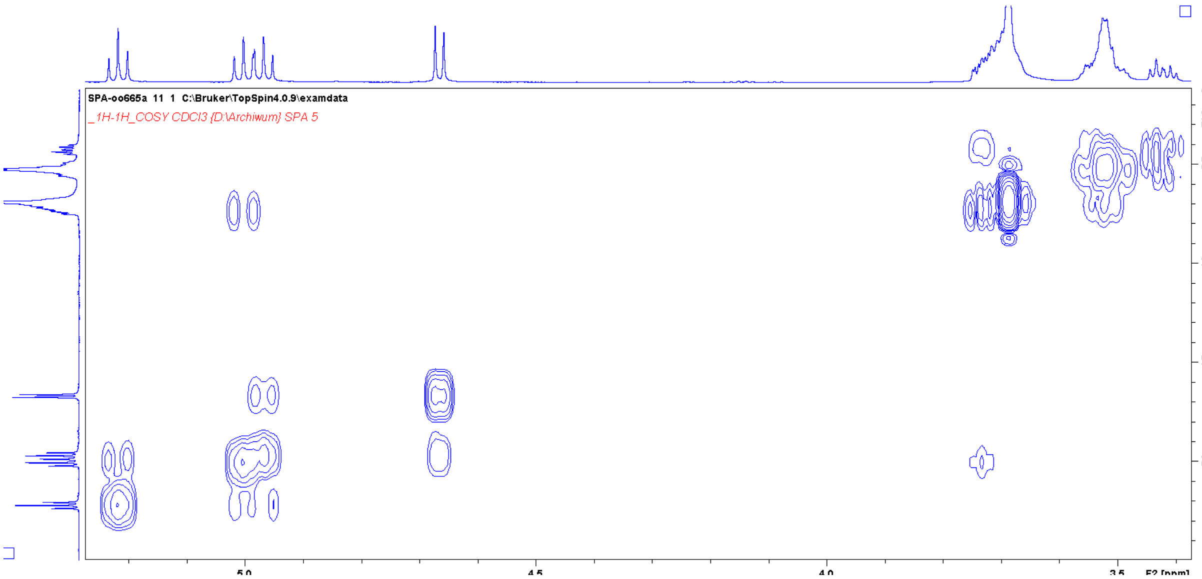

Figure S5. Fragment of COSY spectrum for saccharide and crown ether protons in derivative **7** in $CDCl_3$.

1.2.2. NMR spectra for the cryptand L1, busulfan and lomustine

Cryptand 9 (L1)

NMR ^{1}H (600MHz, DMSO); 7.48(d, 2H, 2NHurea-1, $J_{NH,1}$=8.1); 7.11(d, 2H, 2NH-urea-6, J=8.0); 5.76(d, 2H, 2OH-2, J=5.3); 5.68(d, 2H, 2OH-3, J=6.6); 4.56(d, 2H, 2OH-4, J=4.8);4.56(t, 2H, 2H-1, J=8.5); 3.57-2.98(m, 34H, 2H-2, 2H-3, 2H-4, 2H-5, 2H-6); yield: 16%.

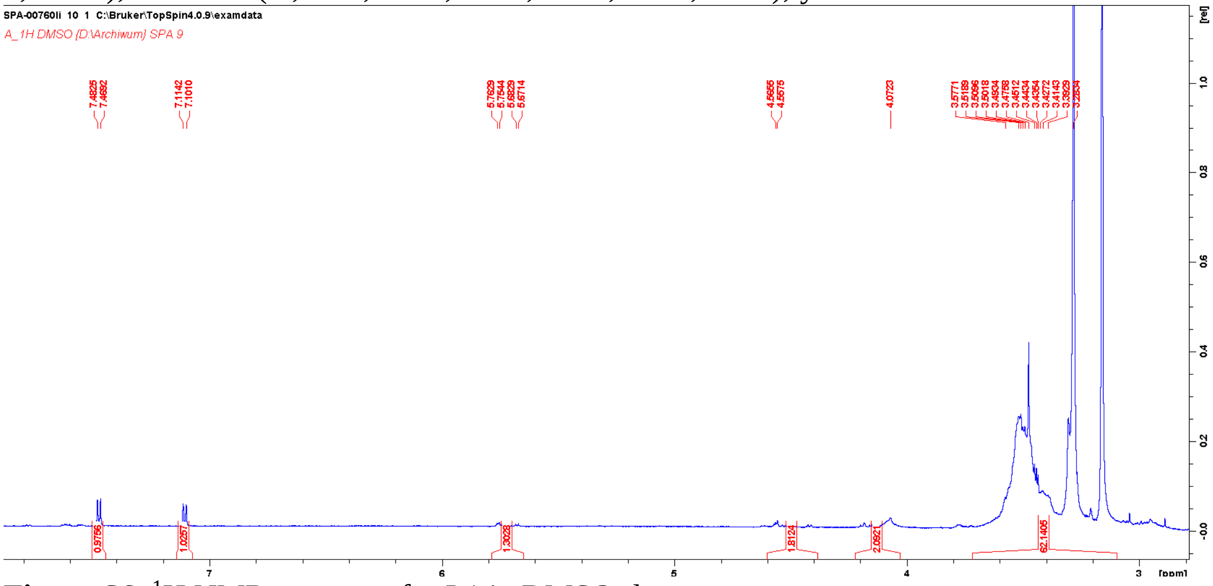

Figure S6. ^{1}H NMR spectrum for L1 in DMSO-d$_6$.

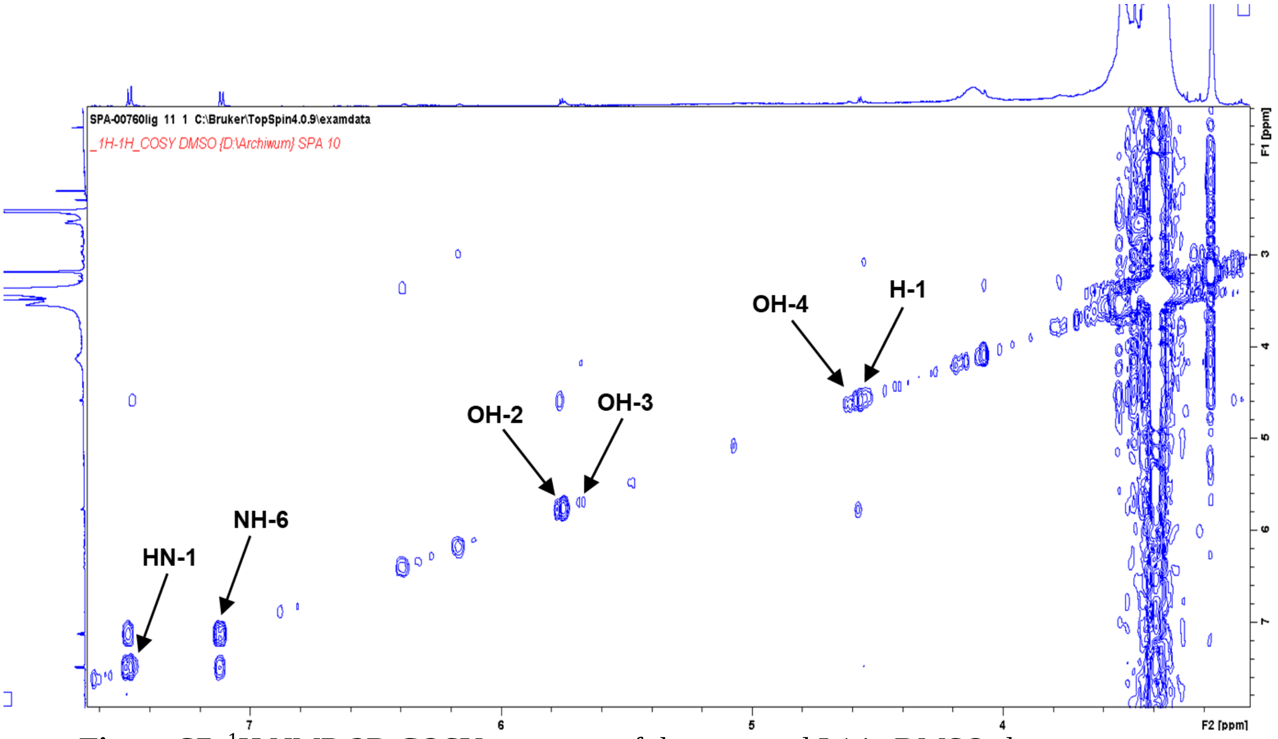

Figure S7. ^{1}H NMR 2D COSY spectrum of the cryptand L1 in DMSO-d$_6$.

Busulfane (BSF)

NMR ^{1}H (600MHz, DMSO); 4.24 (t_{ps}, 4H, $2CH_2$, $\underline{CH_2}N$, J=11.6); 3.17 (s, 6H, $2CH_3$); 1.76 (t_{ps}, 4H, $2CH_2$, $\underline{CH_2}CH_2$)

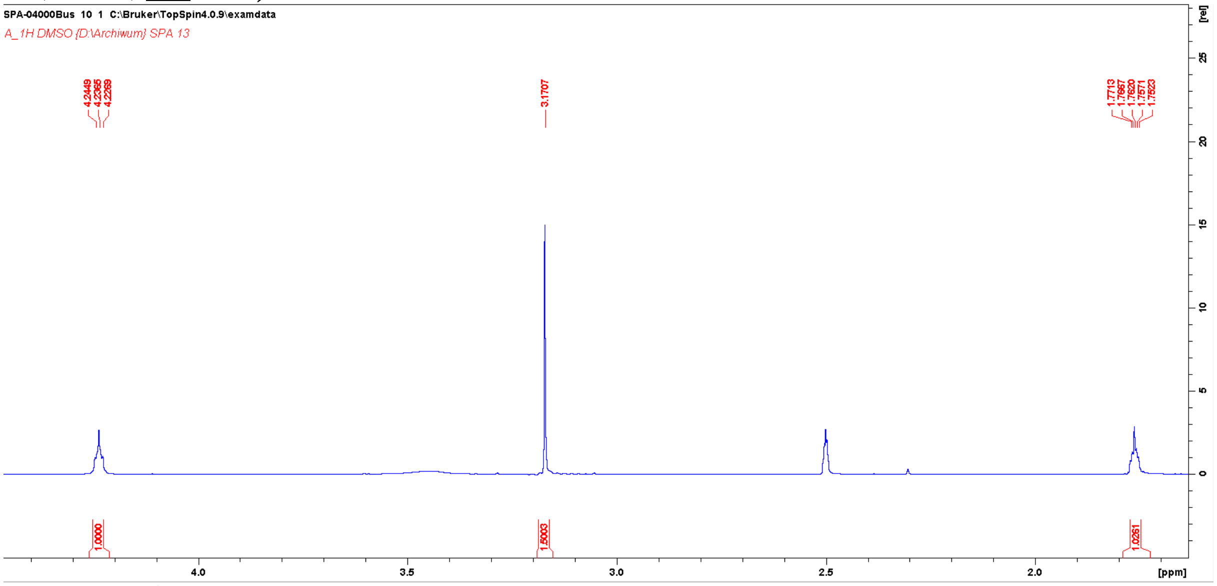

Figure S8. ^{1}H NMR spectrum for BSF in DMSO-d_6.

Lomustine (CCNU)

NMR ^{1}H (600MHz, DMSO); 8.50(d, 1H, NH, J=7.2); 4.08(t, 2H, CH_2, $C\underline{H_2}Cl$, J=5.8); 3.72-3.65(m, 1H, H-1); 3.61(t, 2H, CH_2, $C\underline{H_2}N$, J=5.8); 1.87-1.81(m,2H, H-2a, H-6a); 1.76-1.70(m, 2H, H-3a, H-5a); 1.62-1.57(m, 1H, H-4a); 1.45-1.37(m, 2H, H-2b, H-6b); 1.34-1.25(m, 2H, H-3b, H-6b); 1.14-1.06(m, 1H, H-4b)

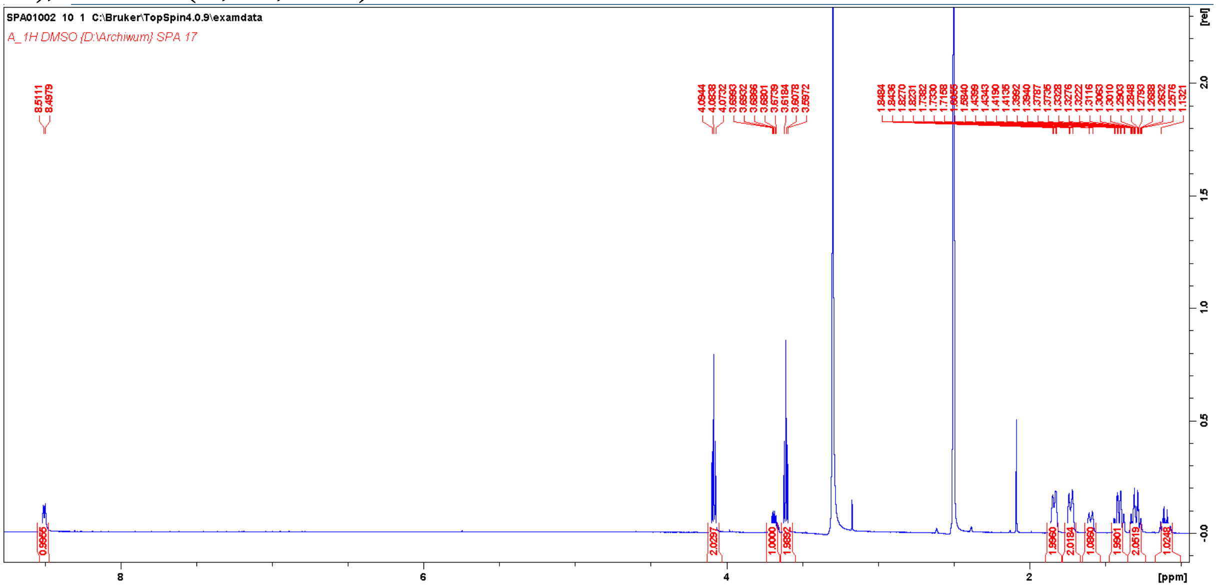

Figure S9. ^{1}H NMR spectrum for CCNU in DMSO-d_6.

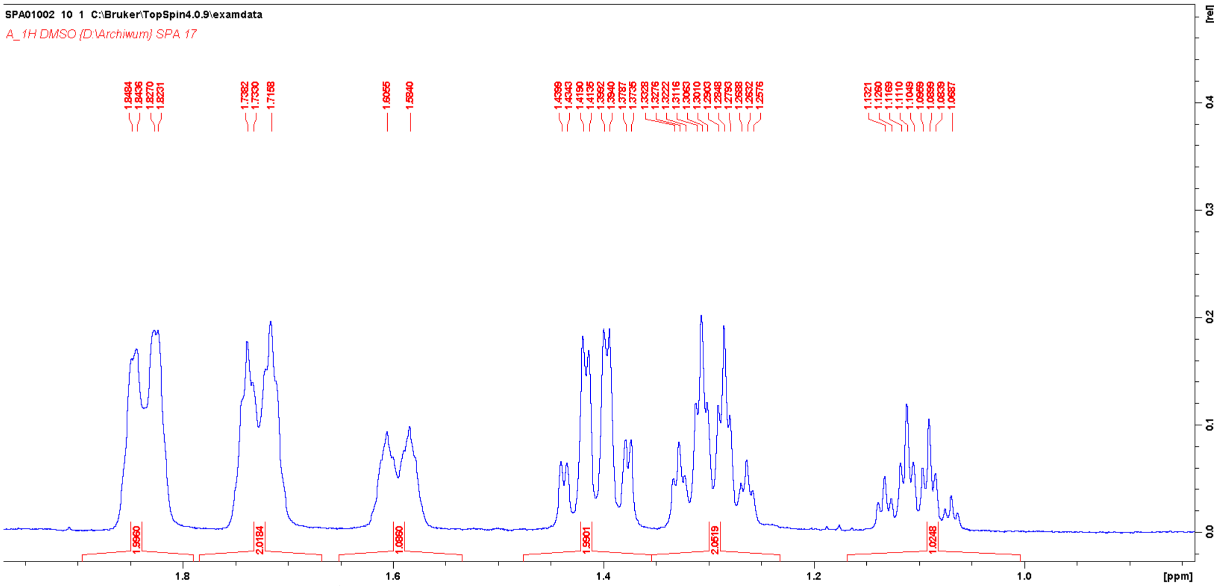

Figure S10. Fragment of ^{1}H NMR spectrum for cyclohexane protons in CCNU in DMSO-d_6.

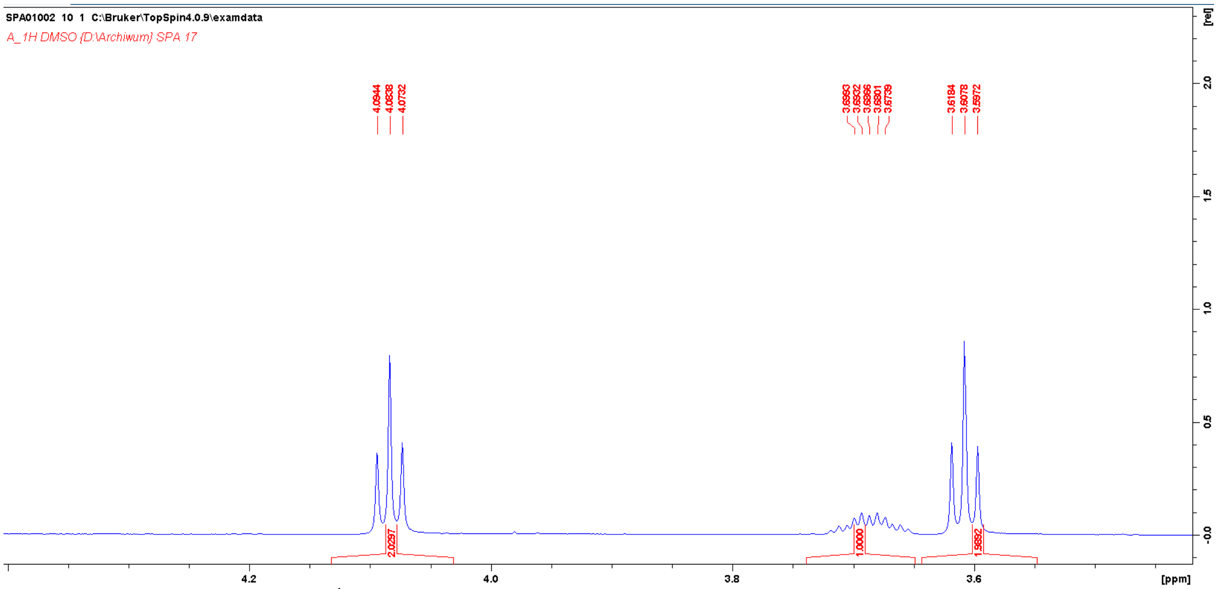

Figure S11. Fragment of ^{1}H NMR spectrum for protons of H-7 and H-8 in CCNU in DMSO-d_6.

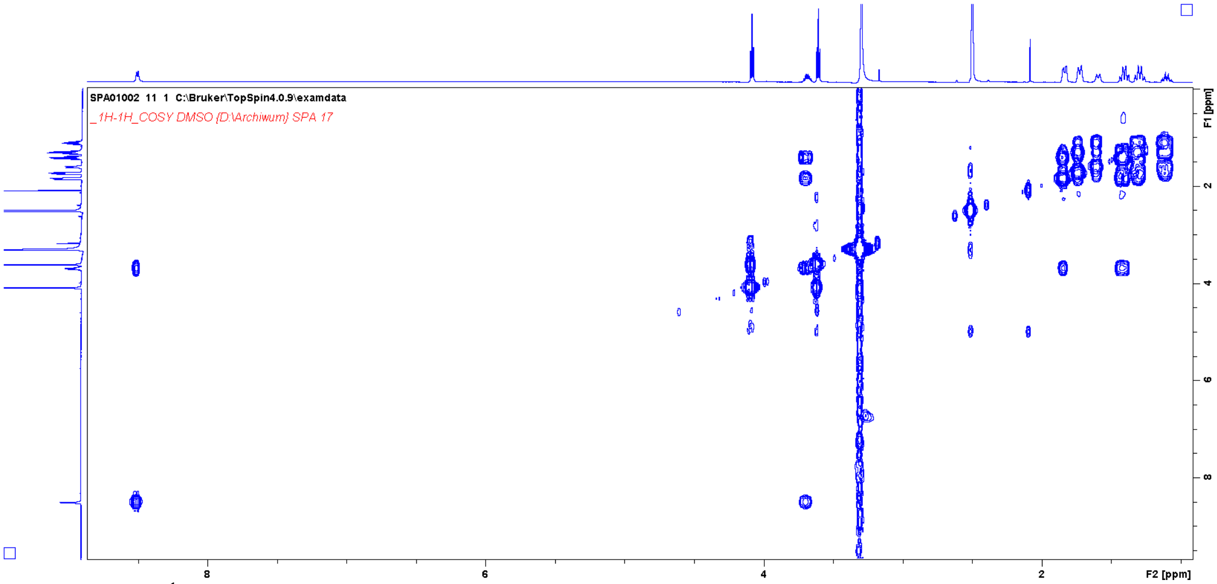

Figure S12. ^{1}H NMR 2D COSY spectrum for CCNU in DMSO-d_6.

1.2.3. Superimposed NMR spectra of L1, drugs and complexes

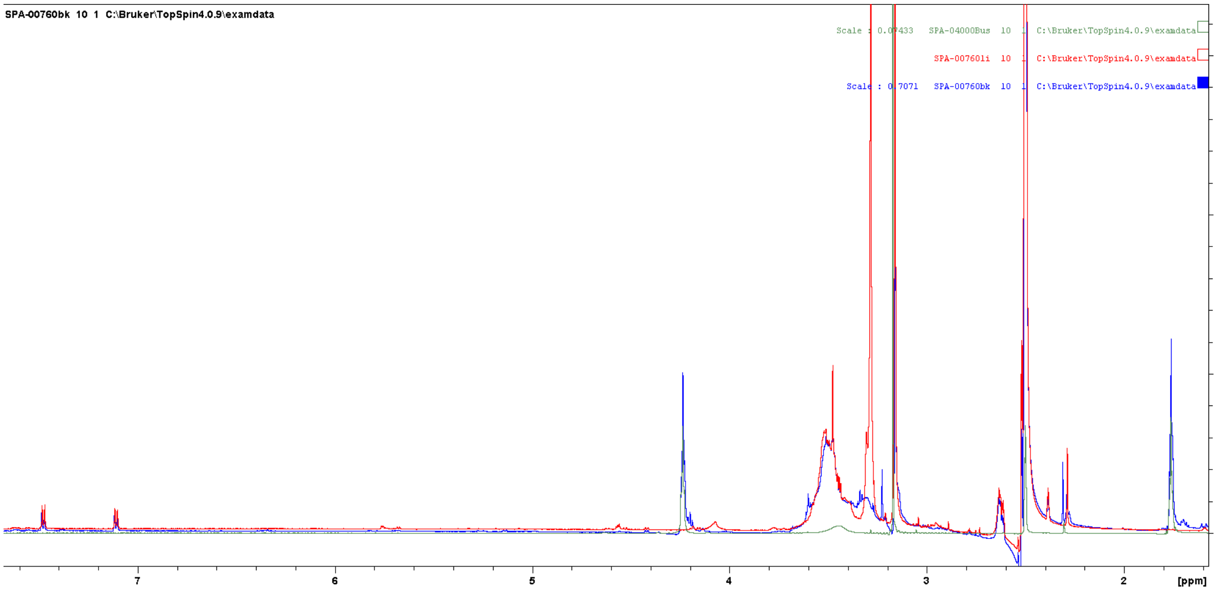

Figure S13. Superimposed ^{1}H NMR spectra of the complex L1:BSF in stechiometry 1:1 (blue), free BSF (green) and free lignad L1 (red) in DMSO-d_6.

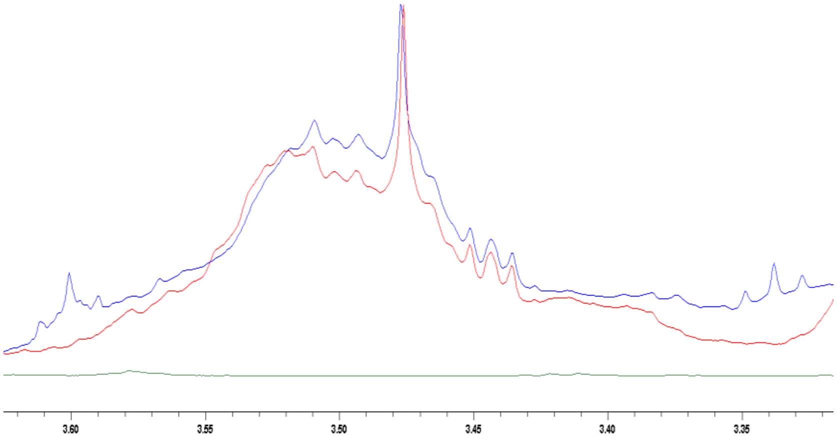

Figure S14. Fragment of superimposed ^{1}H NMR spectra of the complex L1:BSF in stechiometry 1:1 (blue), free BSF (green) and free lignad L1 (red) in DMSO-d_6.

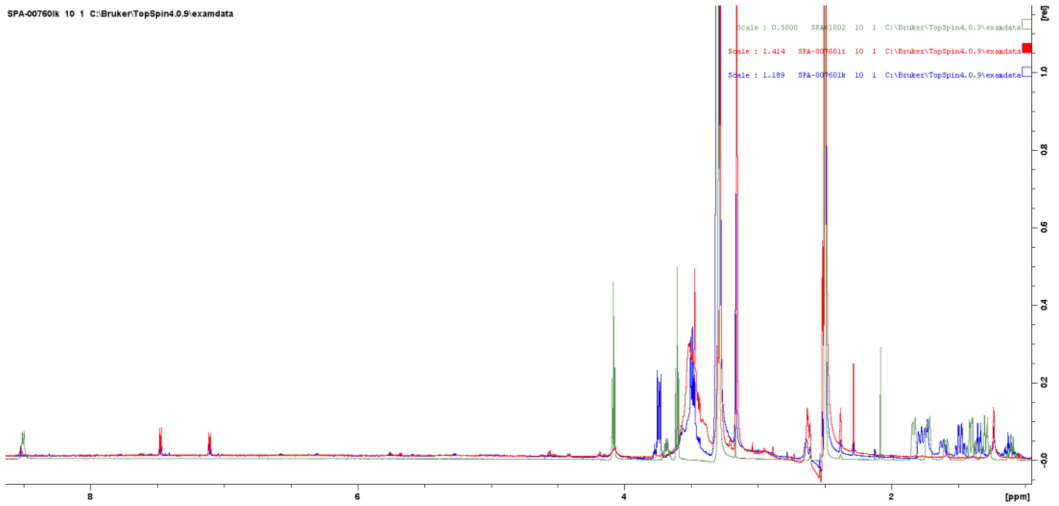

Figure S15. Superimposed ^{1}H NMR spectra of the complex L1:CCNU in stechiometry 1:1 (blue), free CCNU (green) and free lignad L1 (red) in DMSO-d_6.

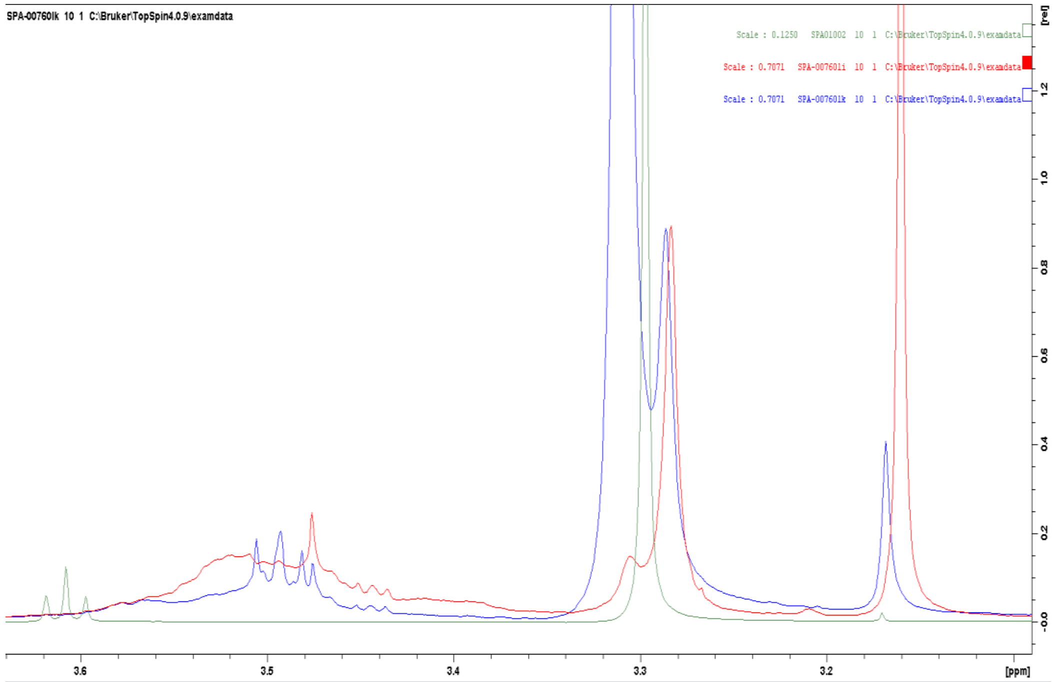

Figure S16. Fragment of superimposed ^{1}H NMR spectra of the complex L1:CCNU in stechiometry 1:1 (blue), free CCNU (green) and free lignad L1 (red) in DMSO-d_6.

2. Theoretical part

2.1. Details of calculation methods

Procedure S1.

Description of the search for the lowest energy conformers of the molecules L1, BSF and CCNU.

The initial structures of the cryptand L1 and the drugs BSF and CCNU, were created in the Hyperchem program.[SR1] In each molecule were pre-defined the most important torsion angles, which are shown in Figure S17. Next, using the molecular mechanics force field Amber99 and the Conformational Search module available in Hyperchem, a large number of structures was created (25880 for L1, 5779 for BSF and 2017 for CCNU) by random varying selected torsion angles in the ranges 0-180° (acyclic) and 0-120° (in rings), and optimized afterwards with the Amber99 force field.

In the second step, all these structures were fully re-optimized in the MOPAC program[SR2] using the semiempirical method PM7 in vacuo. Additionally, for three most stable structures of each drug and ligand L1 found from the PM7 calculations, the semiempirical Stochastic Dynamics simulations with Verlet algorithm[SR3] were performed in the program Gabedit[SR4] using the Conformational Search module combined with MOPAC. From each trajectory 100 lowest energy conformers were selected and optimized with the PM7 method. In each case, the simulation was repeated, using the most stable geometry indicated in the previous one, until no lower energy structure was found.

In the third step, 300 different lowest energy conformers of L1 selected after all semiempirical calculations and all conformers of each drug (excluding duplicates) were fully optimized in the Gaussian 09 program[SR5] using the 6-31G(d,p) basis set and the M06-2X-GD3 method. This is the hybrid metafunctional M06-2X[SR6] with the Grimme empirical pairwise long range (dispersion) corrections GD3.[SR7] All DFT calculations were performed in water, with the solvent being described by the Polarizable Continuum Model (PCM).[SR8] To evaluate an effect of the diffuse functions on the relative energies, for 10 lowest energy structures of the drugs and L1, the single point (SP) calculations were performed with the 6-31++G(d,p) basis set.

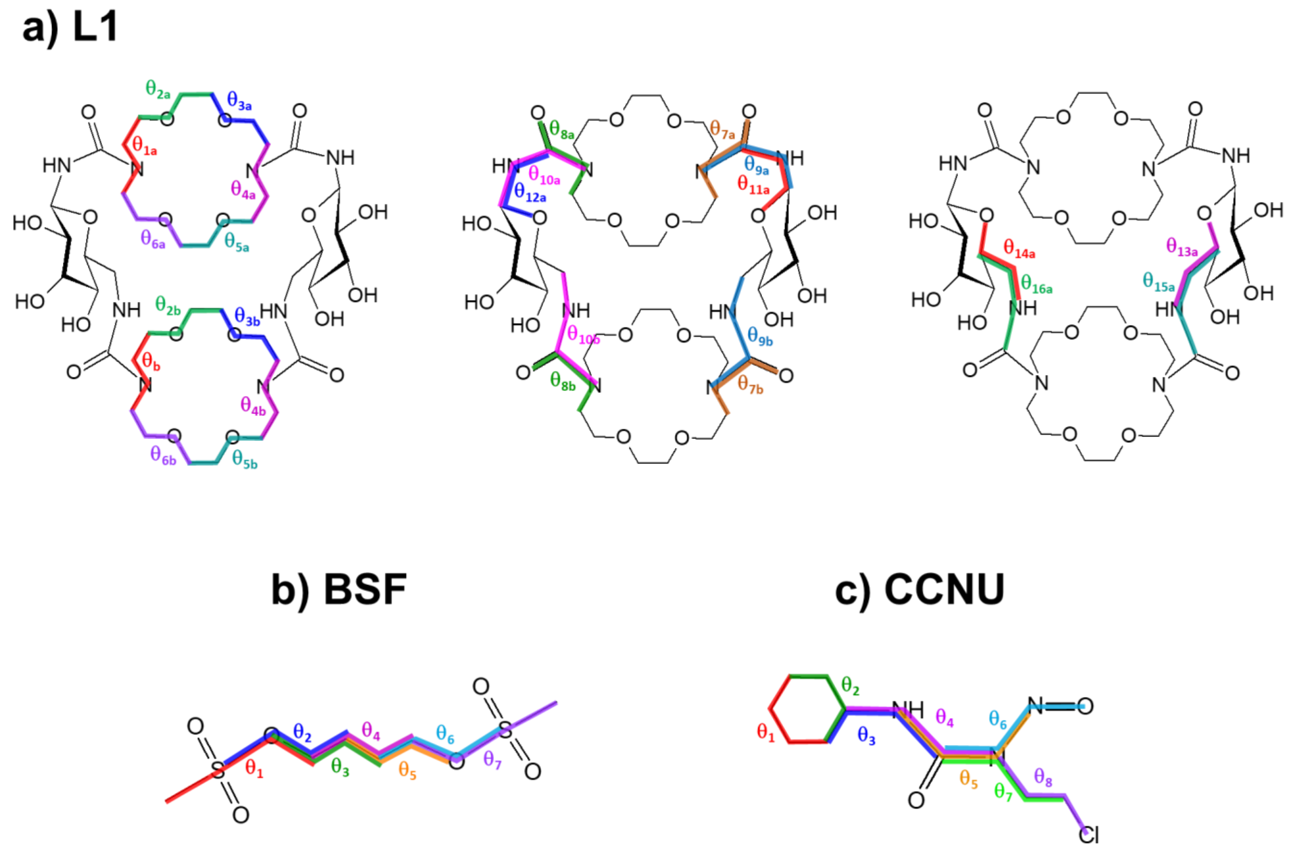

Figure S17. The torsion angles in the molecules: a) the cryptand L1, b) busulfan (BSF), c) lomustine (CCNU), varied in the molecular mechanics conformational search.

Procedure S2.

Description of the search for the lowest energy configurations of L1:BSF and L1:CCNU.

The initial structures of their complexes were created using the most stable structures of L1, BSF and CCNU, found in water at the M06-2X-GD/6-31G(d,p)/PCM theory level. Seven different positions of the drug with respect to L1 were considered, as shown in Figure S17. For six positions A-F, the drug molecule was placed at two different distances from the origin, d_1 and d_2, while in the G configuration it was placed in the center of L1. In the latter case, before any further steps, some adjustment of L1 to the presence of the drug was done by performing 10 cycles of optimization of L1 while the drug structure was kept frozen. Next, for each site and a given distance, a set of 2916 structures were created in the Hyperchem program by rotating the drug molecule around each axis, changing the angles every 20° in the following ranges: 0-180° (X), 0-360° (Y) and 0-360° (Z). The resulting structures were optimized with the method PM7 in vacuo in the MOPAC program. For each configuration A-G, ten lowest energy structures were selected and fully re-optimized with the M06-2X-GD3/6-31G(d,p) method in water (PCM). Additionally, for all resulting structures were performed the M06-2X-GD3 single point calculations with the 6-31++G(d,p) basis set in water. The DFT calculations for the complexes were performed using the Gaussian 09 or 16 packages.[SR5,SR9]

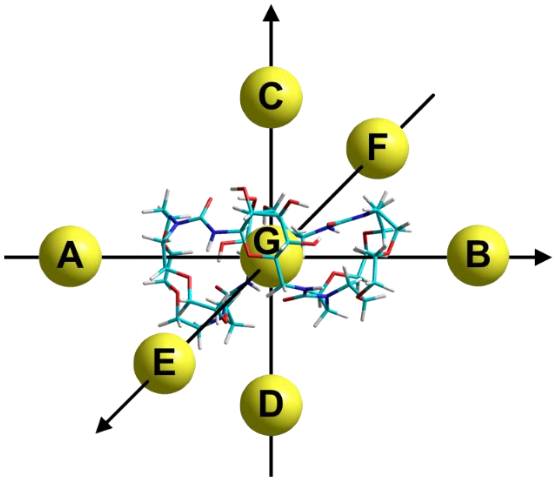

Drug	Position/distance	**A**	**B**	**C**	**D**	**E**	**F**	**G**
BSF	d_1 [Å]	10	11	7	7	8	8	0
	d_2 [Å]	11	12	8	8	9	9	
CCNU	d_1 [Å]	10	11	7	7	8	8	0
	d_2 [Å]	12	13	9	9	10	10	

Figure S18. Seven configurations of the complexes L1:BSF and L1:CCNU considered in the calculations and the distances of BSF and CCNU from the center of L1.

Procedure S3.

The method of creation of the models including 20 H_2O.

For the ligand L1, the models were created separately for each of its three selected structures: the most stable conformer L1_MS and two "open" geometries extracted from the inclusion complexes G of L1:BSF (L1_O1) and L1:CCNU (L1_O2). First, in the Hyperchem program, water molecules were added to L1 in a box of a size 20×20×20Å (210 H_2O for the most stable L1 and for L1 from L1:CCNU, and 208 H_2O for L1 from L1:BSF), Next, the structure of L1 was frozen and, to decrease the gradient, 100 of optimization cycles were conducted for the water molecules in the force field Amber99 using the periodic boundary conditions. The positions of water molecules were then further adjusted in the same force field by performing the Molecular Dynamics simulations of water molecules (L1 was kept frozen). The system was heated for 2ps from 0 to 300K, then the simulation was run for a period of 20ps at the temperature 300K, and finally the system was cooled for another 2ps to 0K. The time step during the simulation was 1fs. After the simulation, the more distant water molecules were removed, leaving the cluster containing L1 and 20 H_2O. The resulting cluster L1:20H_2O was used as the initial geometry for the PM7 semiempirical Stochastic Dynamics simulations with Verlet algorithm, which were performed in the Gabedit program using the Conformational Search module combined with the MOPAC program. In these simulations, the solvent effect was taken into account by using the Conductor-like Screening Model (COSMO).[SR10] Also in these calculations the structure of L1 was frozen. From each trajectory 100 lowest energy conformers were selected and partially optimized (L1 frozen) with the PM7 method in water (COSMO). In each case, the simulation was repeated, using the most stable geometry indicated in the previous one, until no lower energy structure was found.

The procedure applied for the complexes L1:BSF and L1:CCNU was very similar, except that in the Hyperchem program waters were added in a larger box of a size 25×25×25Å. Hence, the number of water molecules added was also higher: 466 for L1:BSF (F), 470 for L1:BSF (G), 466 for L1:BSF (F), 469 for L1:CCNU (C) and 461 for L1: CCNU (G). In the simulations the whole complex, L1:BSF or L1:CCNU, was kept frozen, thus only water molecules were allowed to move.

Finally, for each system separately, from all structures optimized with the PM7 method after simulations, 10 configurations with the lowest energy were selected and fully re-optimized (without any constraints) with the M06-2X-GD3/6-31G(d,p)/PCM method and the most stable configuration was selected.

2.2. Structures and energies of complexes in water (PCM)

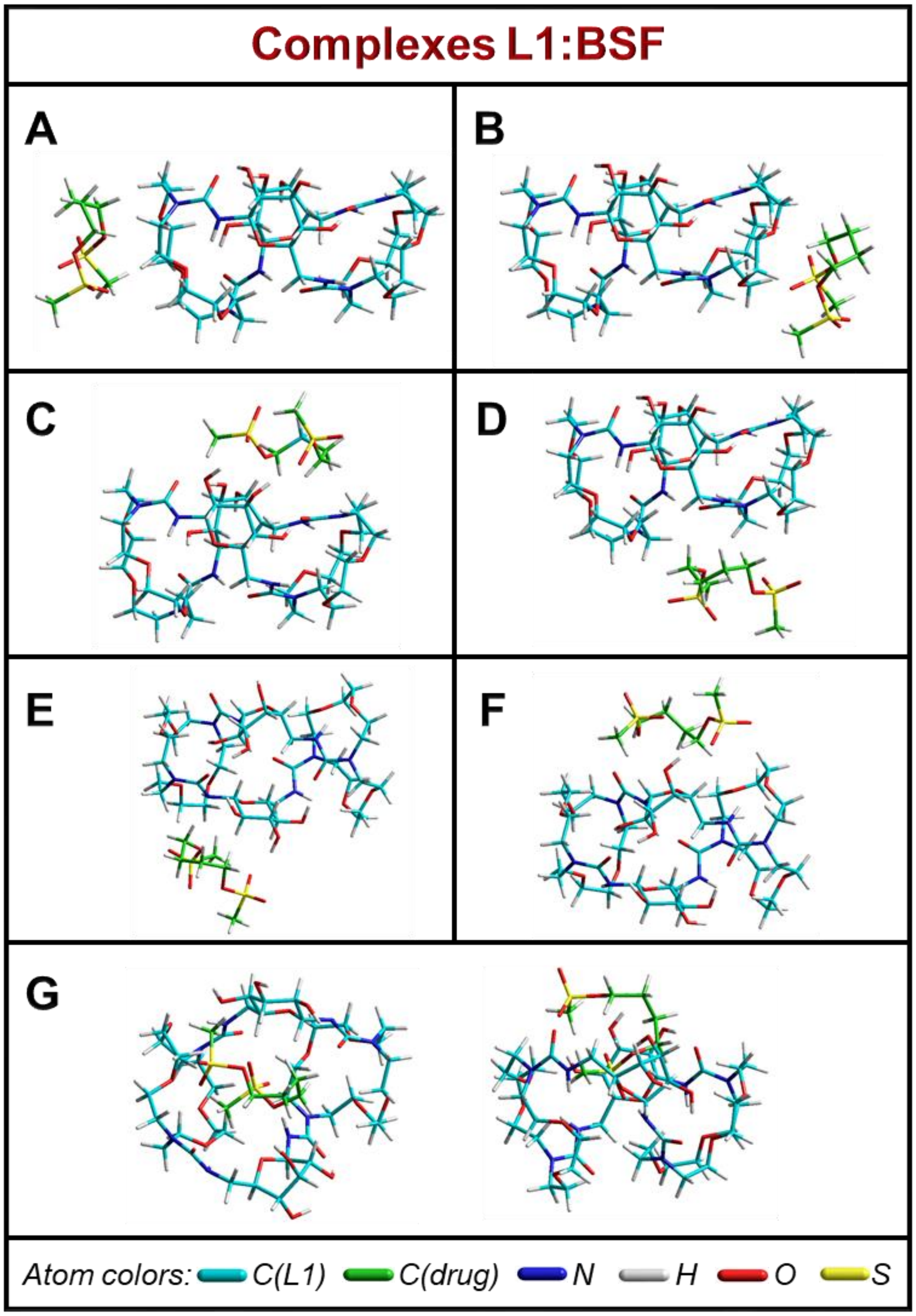

Figure S19. The lowest energy structures of the complex L1:BSF in seven different configurations, obtained from the M06-2X-GD3/6-31G(d,p) optimizations in water (PCM) performed at the final step of the configurational search. To distinguish the two molecules forming the complex, different colors were used for the carbon atoms in BSF (green) and in L1 (cyan). For the complexes E and F, their "top" view is presented; for the inclusion complex G both "top" and "side" views are shown.

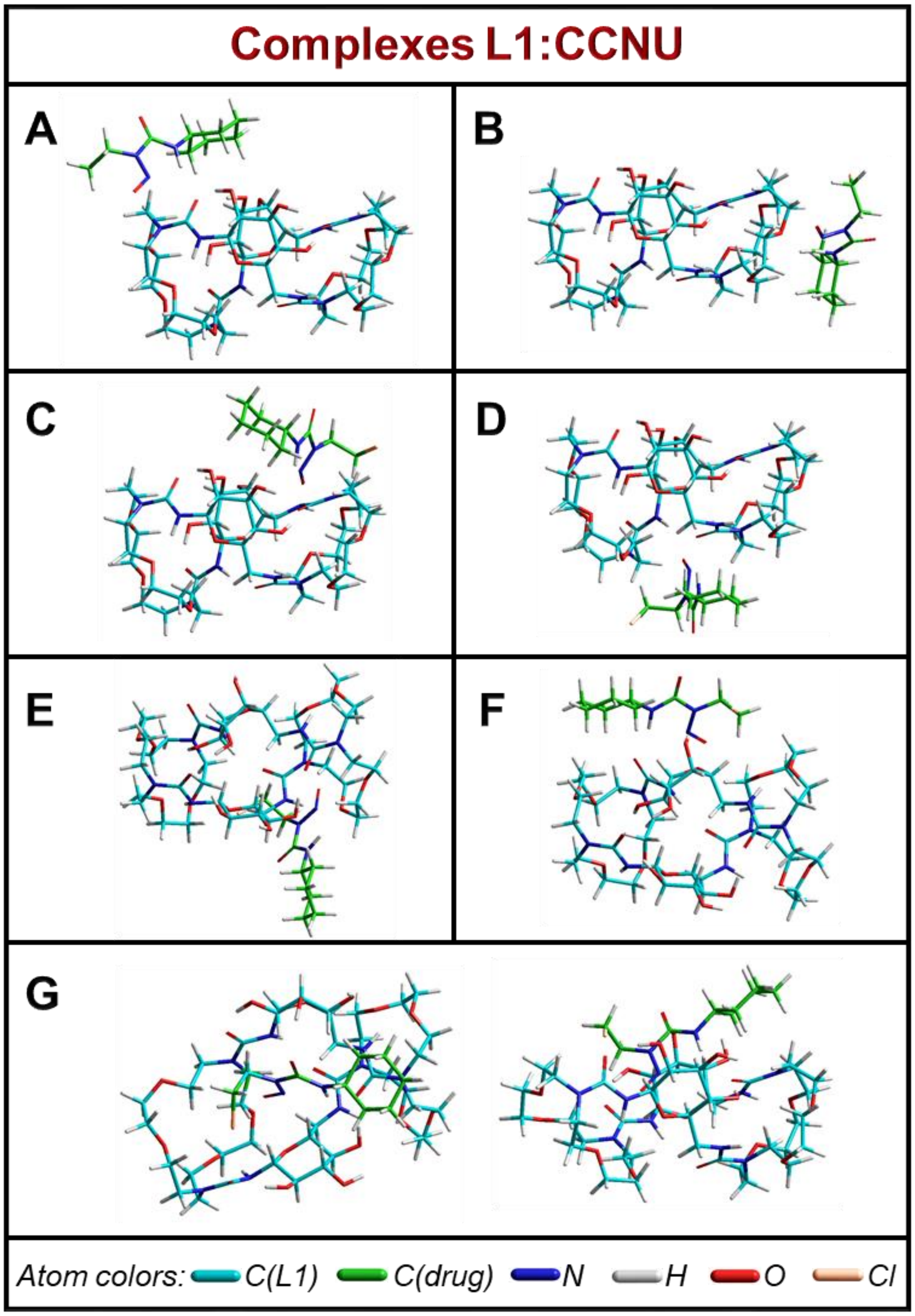

Figure S20. The lowest energy structures of the complex L1:CCNU in seven different configurations,, obtained from the M06-2X-GD3/6-31G(d,p) optimizations in water (PCM) performed at the final step of the configurational search. To distinguish the two molecules forming the complex, different colors were used for the carbon atoms in BSF (green) and in L1 (cyan). For the complexes E and F, their "top" view is presented; for the inclusion complex G both "top" and "side" views are shown.

Table S1. The total energies *E*, enthalpies *H* and corrected Gibbs energies G_{corr} obtained from the M06-2X-GD3/6-31G(d,p) optimization (OPT) in water (PCM) for the lowest energy structures BSF, CCNU, L1 and seven configurations of the complexes L1:BSF and L1:CCNU. In the last column are given the energies *E* obtained from the single point (SP) calculations performed with the 6-31++G(d,p) basis set for the structures optimized with the 6-31G(d,p) basis set. All values are in hartree.

**Structure**	**OPT 6-31G(d,p)** ***E***	**OPT 6-31G(d,p)** ***H***	**OPT 6-31G(d,p)** G_{corr}	**SP 6-31++G(d,p)** ***E***
**BSF**	-1484.34625869	-1484.106489	-1484.152375	-1484.36952794
**CCNU**	-1127.17127073	-1126.892518	-1126.938049	-1127.19077444
**L1**	-3508.88566935	-3507.599404	-3507.737876	-3508.99286465
**L1:BSF A**	-4993.25105515	-4991.722789	-4991.894166	-4993.37914195
**L1:BSF B**	-4993.25696444	-4991.728612	-4991.899686	-4993.38548490
**L1:BSF C**	-4993.26384019	-4991.735391	-4991.905830	-4993.39244154
**L1:BSF D**	-4993.26082243	-4991.732313	-4991.902830	-4993.38806023
**L1:BSF E**	-4993.26020686	-4991.731316	-4991.900908	-4993.38730212
**L1:BSF F**	-4993.26739283	-4991.738651	-4991.909050	-4993.39275903
**L1:BSF G**	-4993.24727154	-4991.72004	-4991.890095	-4993.37458709
**L1:CCNU A**	-4636.08642944	-4634.518776	-4634.689636	-4636.20914832
**L1:CCNU B**	-4636.08165834	-4634.514154	-4634.685068	-4636.20645214
**L1:CCNU C**	-4636.09847825	-4634.530695	-4634.699264	-4636.21781698
**L1:CCNU D**	-4636.09300137	-4634.525032	-4634.692982	-4636.21525665
**L1:CCNU E**	-4636.08727683	-4634.519882	-4634.687641	-4636.20850962
**L1:CCNU F**	-4636.09388939	-4634.526743	-4634.696194	-4636.21662208
**L1:CCNU G**	-4636.07129514	-4634.504234	-4634.675021	-4636.19639262

Table S2. The complexation energies (E_{compl}), enthalpies (H_{compl}) and corrected Gibbs energies (G_{corr_compl}) obtained for different configurations of L1:BSF and L1:CCNU from the M06-2X-GD3/6-31G(d,p) optimizations (OPT; BSSE corrected) in water (PCM) as well as selected results from the single point calculations (SP; without the BSSE corrections) performed with the 6-31++G(d,p) basis set. For the OPT series, the interaction energies (E_{int}) and deformation energies for each drug (E_{def_drug}) and L1 (E_{def_L1}) are additionally included. All values are in kcal/mol.

	**OPT BSSE 6-31G(d,p)**						**SP 6-31++G(d,p)**		
**Structure**	E_{compl}	H_{compl}	G_{corr_compl}	E_{int}	E_{def_drug}	E_{def_L1}	E_{compl}	H_{compl}	G_{corr_compl}
**L1:BSF A**	-9.5	-8.1	0.1	-12.9	0.2	0.7	-10.5	-9.1	-1.0
**L1:BSF B**	-13.7	-12.2	-3.9	-16.7	0.5	0.4	-14.5	-13.0	-4.7
**L1:BSF C**	-17.3	-15.8	-7.1	-28.3	2.9	5.4	-18.9	-17.3	-8.6
**L1:BSF D**	-14.0	-12.5	-3.8	-19.1	0.2	0.8	-16.1	-14.6	-5.9
**L1:BSF E**	-13.8	-12.0	-2.8	-21.6	0.9	2.9	-15.6	-13.8	-4.6
**L1:BSF F**	-18.2	-16.5	-7.8	-25.7	3.2	0.2	-19.1	-17.4	-8.6
**L1:BSF G**	-1.8	-1.1	7.9	-42.7	2.5	30.6	-7.7	-6.9	2.1
**L1:CCNU A**	-14.0	-12.4	-4.1	-20.0	1.1	0.4	-16.0	-14.4	-6.1
**L1:CCNU B**	-13.0	-11.4	-3.2	-17.1	1.3	0.3	-14.3	-12.8	-4.5
**L1:CCNU C**	-19.8	-18.1	-8.4	-28.7	1.8	0.9	-21.4	-19.7	-10.0
**L1:CCNU D**	-17.2	-15.4	-5.3	-25.4	1.4	1.4	-19.8	-18.0	-7.9
**L1:CCNU E**	-14.5	-13.0	-2.8	-22.0	1.3	1.6	-15.6	-14.1	-3.9
**L1:CCNU F**	-18.8	-17.5	-8.3	-24.7	0.7	0.7	-20.7	-19.4	-10.2
**L1:CCNU G**	-1.0	0.3	8.6	-36.4	1.6	25.8	-8.0	-6.7	1.6

2.3. Computed ^{1}H NMR chemical shifts

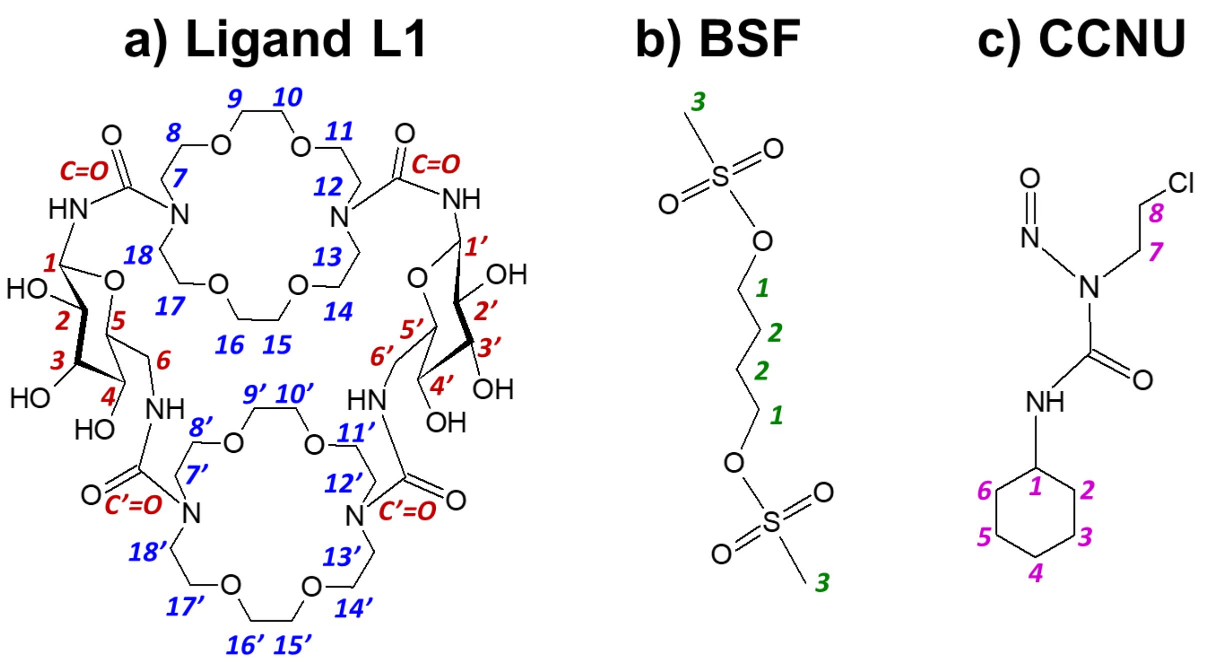

Figure S21. The carbon atom numbers in the molecules L1, BSF and CCNU, used in labels of protons in the ^{1}H NMR analysis of chemical shifts.

Table S3. The averaged ^{1}H NMR chemical shifts δ [ppm] and their change Δδ after complexation, obtained from the M06-2X/6-31++G(d,p)//M06-2X-GD3/6-31G(d,p) calculations in DMSO (PCM) for selected atom types in the free molecules (BSF, CCNU, L1_MS) and in the complexes L1:BSF (configurations F and G) and L1:CCNU (configurations C and G). The averages are calculated from δ values for equivalent protons present in a given molecule (eg. NH-1 and NH-1' in L1, Figure S21a), hence proton labels indicate only the general type of atoms. For some protons in CCNU, a and b indicate higher and lower δ values, respectively.

	δ					Δδ			
Proton label	Free molecule	L1:BSF F	L1:BSF G	L1:CCNU C	L1:CCNU G	L1:BSF F	L1:BSF G	L1:CCNU C	L1:CCNU G
**L1**									
NH-1	6.9529	6.8125	6.2793	6.8973	5.3389	-0.1404	-0.6736	-0.0556	-1.6140
NH-6	6.8847	6.7849	6.5337	6.5614	6.5225	-0.0998	-0.3510	-0.3233	-0.3622
OH-2	2.9756	2.9732	5.5092	2.9354	5.4824	-0.0024	2.5336	-0.0401	2.5068
OH-3	4.0755	4.0416	2.6772	4.1884	2.3518	-0.0339	-1.3983	0.1129	-1.7238
OH-4	3.5781	4.3452	2.9564	2.9129	4.0969	0.7671	-0.6217	-0.6652	0.5188
H-1	4.9313	5.2731	4.9374	4.9135	4.0810	0.3418	0.0061	-0.0179	-0.8504
H-2	3.0670	3.0753	3.2220	3.0295	2.5451	0.0084	0.1550	-0.0375	-0.5218
H-3	3.5424	3.5528	3.8887	3.4873	2.9621	0.0105	0.3463	-0.0551	-0.5802
H-4	3.1261	3.1180	3.1492	3.1522	2.7013	-0.0080	0.0231	0.0262	-0.4248
H-5	3.5130	3.7232	3.9754	3.6695	3.8494	0.2103	0.4624	0.1565	0.3365
H-6	3.2895	3.3482	3.3439	3.2420	3.2639	0.0588	0.0544	-0.0475	-0.0255
**BSF**									
H-1	4.0886	4.4584	4.2198			0.3698	0.1313		
H-3	3.0120	3.1743	3.5581			0.1624	0.5461		
H-2	1.8532	1.7843	2.1464			-0.0689	0.2932		
**CCNU**									
HN	6.5356			7.4192	7.7197			0.8836	1.1841
H-8	3.3527			3.4132	3.2207			0.0605	-0.1320
H-1	3.4735			3.2084	3.4977			-0.2651	0.0243
H-7	3.9213			3.9752	4.1865			0.0539	0.2652
H-2a, H-6a	2.1025			1.8732	2.0419			-0.2294	-0.0606
H-3a, H-5a	1.7519			2.1259	1.7109			0.3740	-0.0410
H-4a	1.6141			1.6422	1.5550			0.0282	-0.0590
H-2b, H-6b	1.2613			1.5919	1.5183			0.3306	0.2570
H-3b, H-5b	1.4542			1.2729	1.4623			-0.1813	0.0082
H-4b	1.2739			1.4125	1.2018			0.1385	-0.0721

Table S4. The computed ^{1}H NMR chemical shifts δ [ppm] for individual protons of L1 alone and in complexes (configurations F and G) and L1:CCNU (configurations C and G), obtained from the M06-2X/6-31++G(d,p)//M06-2X-GD3/6-31G(d,p) calculations in DMSO (PCM). δ are scaled according to the procedure of Tantillo,[SR11] using the formula δ=(I–σ)/(–S), where σ are the isotropic values obtained from DFT calculations, and I and S are the scaling factors obtained for this method (their values are given in the main article). The first column contains the atom numbers in the complex, while the second column contains proton labels based on carbons numbering shown in Figure S21.

Atom no.	Proton label	L1 alone (L1_MS)	L1:BSF F	L1:BSF G	L1:CCNU C	L1:CCNU G
86	NH-1	7.1143	6.9612	6.7489	7.2888	6.5932
87	NH -1'	6.7915	6.6638	5.8097	6.5057	4.0845
139	NH -6	6.9230	6.8570	6.9271	6.8112	6.1003
138	NH -6'	6.8463	6.7127	6.1403	6.3115	6.9447
56	OH-2	3.9878	4.0038	5.3236	3.9034	5.5260
37	OH-2'	1.9634	1.9426	5.6947	1.9675	5.4387
55	OH-3	2.7543	2.7365	2.5639	2.8332	3.0319
36	OH-3'	5.3968	5.3466	2.7905	5.5436	1.6716
140	OH-4	4.8027	4.7926	1.9508	4.9233	5.5566
89	OH-4'	2.3535	3.8977	3.9620	0.9025	2.6372
52	H-1	5.0034	5.2082	5.0618	5.0511	4.0576
33	H-1'	4.8593	5.3380	4.8130	4.7758	4.1044
53	H-2	2.9494	2.9304	2.9101	2.9227	2.9285
34	H-2'	3.1845	3.2202	3.5338	3.1363	2.1618
54	H-3	3.6250	3.6464	4.2614	3.6757	2.9544
35	H-3'	3.4598	3.4592	3.5159	3.2989	2.9699
50	H-4	2.7519	2.7460	3.0357	2.7703	2.7437
31	H-4'	3.5002	3.4901	3.2626	3.5342	2.6589
51	H-5	3.3933	3.4019	4.2069	3.4783	3.7632
32	H-5'	3.6326	4.0445	3.7439	3.8607	3.9357
57	H-6	3.9165	4.0645	4.2999	3.9140	4.5789
88	H-6	2.7910	2.8782	2.7469	2.8339	2.7170
38	H-6'	3.5865	3.6786	2.6922	3.5331	2.6559
39	H-6'	2.8639	2.7716	3.6365	2.6869	3.1040
70	H-7	2.7332	2.7494	2.7393	2.7282	2.7354
71	H-7	3.5376	3.5525	3.7177	3.5285	3.5243
122	H-7'	2.9932	2.9593	3.4454	2.9527	2.9354
123	H-7'	2.9665	2.9588	2.5773	2.8305	2.9867
80	H-12	2.4718	2.4446	2.7670	2.4985	2.6636
81	H-12	4.2554	4.3475	3.4451	4.2402	4.3793
132	H-12'	3.3889	3.3383	3.1693	3.5250	3.4847
133	H-12'	3.1412	3.1494	2.8649	3.0566	3.3843
19	H-13	4.2952	4.2914	4.2860	4.2826	3.4882
20	H-13	2.3949	2.3121	2.4807	2.3320	3.0762
108	H-13'	4.4749	4.3124	4.2761	4.3361	4.1910
109	H-13'	2.2411	2.3073	2.3168	2.2801	2.6428
9	H-18	3.8592	3.9181	3.8362	3.8630	3.5478

10	H-18	2.5454	2.4938	2.5516	2.4704	2.5853
98	H-18'	3.0828	3.0936	3.5337	2.9744	2.9021
99	H-18'	3.2331	3.1606	3.6951	3.3371	3.0611
72	H-8	4.9036	4.9959	5.0244	4.8969	4.5119
73	H-8	2.7867	2.7351	2.8754	2.7336	3.0386
124	H-8'	2.9639	3.0433	3.4261	2.9550	3.1756
125	H-8'	5.1150	5.1358	3.3453	5.2804	4.0795
78	H-11	4.2881	4.3905	3.4647	4.3137	4.8793
79	H-11	3.8374	3.8943	3.8683	3.7642	3.9710
130	H-11'	3.4401	3.4568	3.6682	3.2627	3.4535
131	H-11'	3.4057	3.4778	3.5703	3.4172	3.3951
17	H-14	3.1165	3.1177	2.9161	3.0616	3.6880
18	H-14	3.6247	3.6871	4.4091	3.6159	3.8347
106	H-14'	3.0062	2.9030	3.1500	3.0300	3.0452
107	H-14'	3.5603	3.5071	3.5904	3.5620	3.6164
11	H-17	3.8305	3.8968	3.2106	3.7732	3.9356
12	H-17	2.9117	2.8873	3.2718	2.8527	2.6731
100	H-17'	3.5599	3.6309	3.3986	3.5644	3.4370
101	H-17'	3.4629	3.4643	3.5876	3.4300	3.5186
74	H-9	3.3755	3.3746	3.4132	3.3546	3.9641
75	H-9	3.5308	3.6144	3.5184	3.5796	3.3740
126	H-9'	2.6750	2.7257	3.1164	2.6753	2.7631
127	H-9'	3.9908	4.0338	3.6119	3.9323	3.7323
76	H-10	3.4096	3.3554	3.4751	3.2144	3.4709
77	H-10	3.9929	4.0589	3.9466	3.9344	4.1776
128	H-10'	3.7770	4.4531	3.3502	3.7486	3.7329
129	H-10'	3.2340	3.0275	4.3112	2.9949	3.2385
15	H-15	3.5162	3.4841	3.7404	3.5312	3.8054
16	H-15	3.2636	3.2077	3.1326	3.3388	3.5572
104	H-15'	2.7434	2.6192	3.4886	2.7662	2.7148
105	H-15'	4.0841	4.1623	4.2568	4.1180	4.8954
13	H-16	3.4256	3.5124	2.9783	3.5067	3.4235
14	H-16	3.0269	2.9895	3.5068	2.9808	3.3048
102	H-16'	3.4098	3.3990	3.2539	3.4224	3.4187
103	H-16'	3.5774	3.5244	3.2878	3.5171	3.5279

Table S5. The DFT computed, scaled ^{1}H NMR chemical shifts δ [ppm] for individual protons of BSF alone and in the complex L1:BSF (configurations F and G). The atom numbers (first column) are given for BSF in the complex.

Atom no.	Proton label	BSF alone	L1:BSF F	L1:BSF G
155	H-1	3.6395	4.4011	4.3032
156	H-1	4.5872	4.2475	4.7231
161	H-1	3.8846	4.4279	3.7596
162	H-1	4.2430	4.7569	4.0934
157	H-2	1.5279	1.2699	1.8123
158	H-2	2.2171	1.9563	2.8806
159	H-2	1.7168	1.9302	1.8975
160	H-2	1.9511	1.9808	1.9953
163	H-3	2.7428	2.8974	3.2003
164	H-3	3.6533	3.7481	4.0056
165	H-3	2.6848	2.8129	2.5120
166	H-3	2.5783	2.5945	3.9651
167	H-3	2.6559	4.4279	4.1236
168	H-3	3.7567	2.5651	3.5417

Table S6. The DFT computed, scaled ^{1}H NMR chemical shifts δ [ppm] for individual protons of CCNU alone and in the complex L1:CCNU (configurations C and G). The atom numbers (first column)are given for BSF in the complex. For some equivalent protons, the higher and lower δ values, taken for appropriate averages a and b in Table S5 above, are highlighted in blue and orange, respectively

Atom no.	Proton label	CCNU alone	L1:BSF F	L1:BSF G
167	NH	6.5356	7.4192	7.7197
170	H-8	3.2698	3.2991	3.3034
171	H-8	3.4355	3.5273	3.1380
168	H-7	3.7041	3.8607	3.4959
169	H-7	4.1385	4.0896	4.8771
160	H-1	3.4735	3.2084	3.4977
166	H-2a	1.9123	2.0910	1.8648
165	H-2b	1.3542	1.8041	1.7555
164	H-3a	1.7922	1.6551	1.6611
163	H-3b	1.4603	1.2787	1.4409
162	H-4a	1.6141	1.4125	1.5550
161	H-4b	1.2739	1.6422	1.2018
157	H-5a	1.7116	2.5968	1.7608
156	H-5b	1.4480	1.2670	1.4838
159	H-6a	2.2928	1.3797	2.2190
158	H-6b	1.1683	1.6553	1.2810

2.4. Structures and energies of L1, L1:BSF and L1:CCNU with 20 H_2O

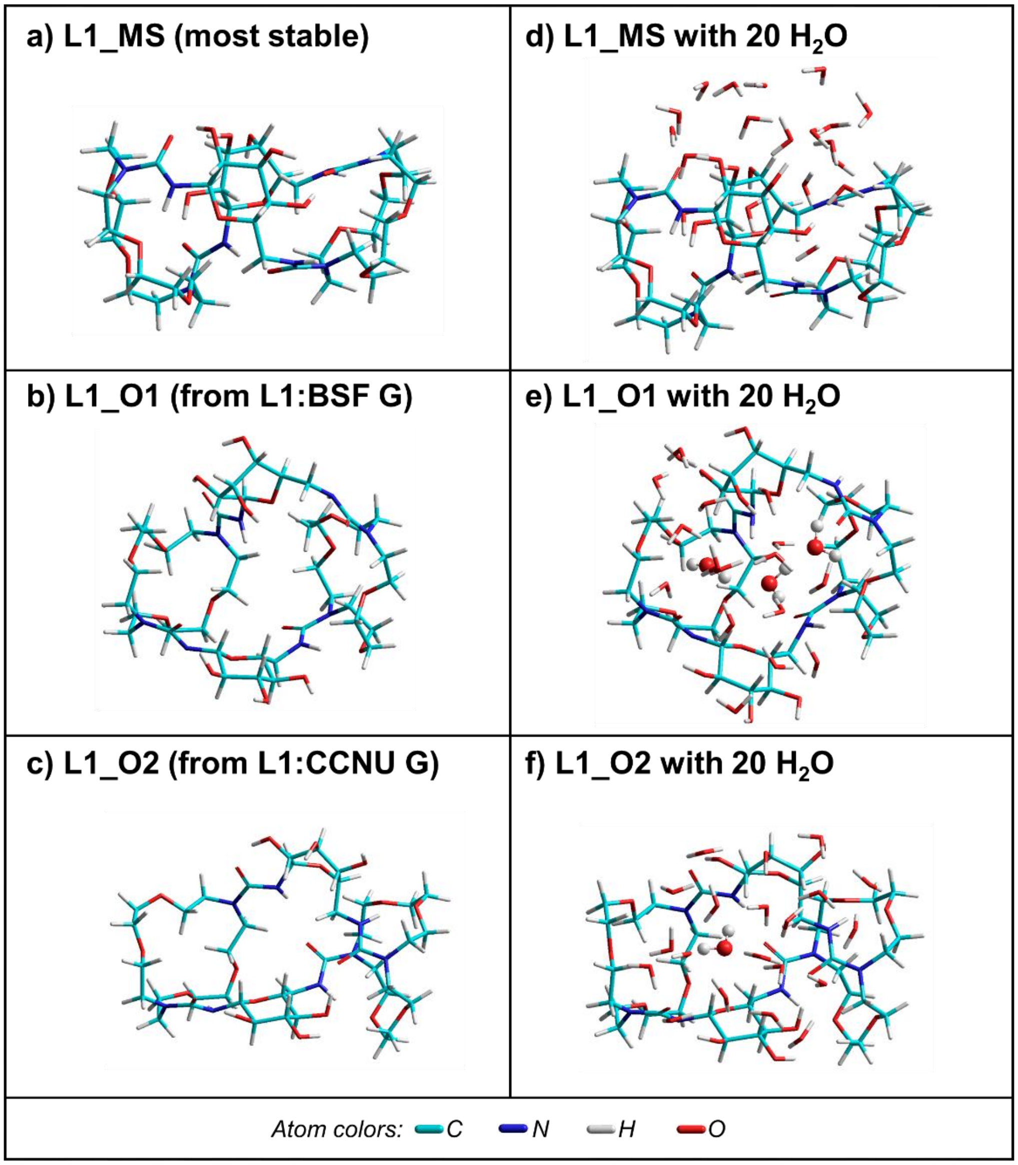

Figure S22. A comparison of geometries of the lowest energy structure L1 (a) and its two "open" structures, taken from the G configurations of the complexes L1:BSF (b) and L1:CCNU (c) obtained in water (PCM) with the corresponding structures obtained in the presence of 20 water molecules after their full optimization (d-f) in water (PCM). For the most stable L1 (a,d) the "side" view is shown, while for the remaining structures (b,c,e,f) it is the "top" view. In e) and f), the water molecules located inside the cavity of L1 are rendered with spheres and cylinders.

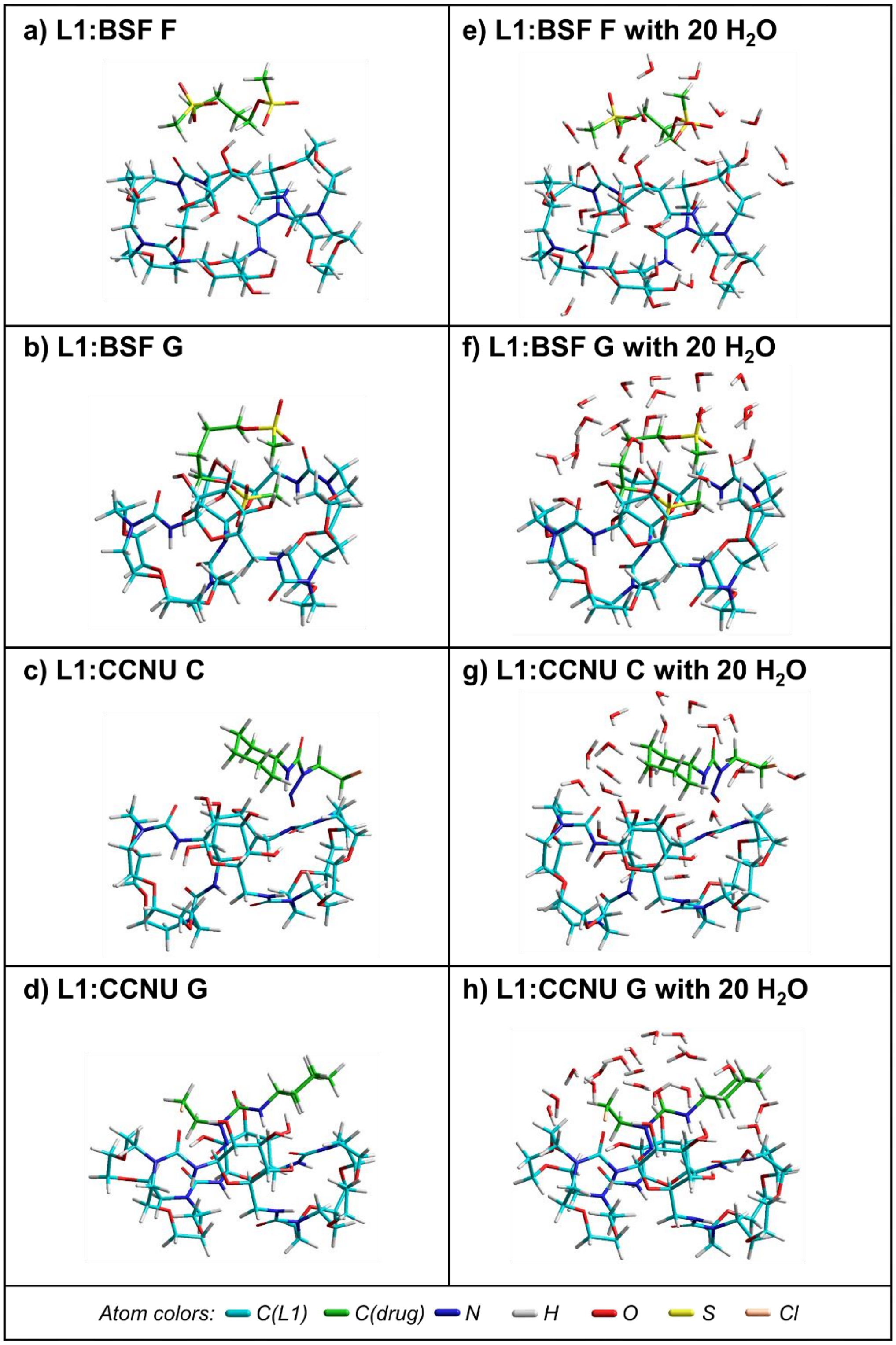

Figure S23. A comparison of geometries of L1:BSF and L1:CCNU in their most stable configurations (a,c) and the inclusion complexes (b,d) obtained in water (PCM) with the corresponding structures obtained in the presence of 20 water molecules after their full optimization (e-h) in water (PCM).

Table S7. The total energies (E) and relative energy differences (ΔE_{rel}) for selected structures shown in Figures S20 and S21, obtained with the M06-2X-GD3/6-31G(d,p) method. The relative energy difference was calculated with respect to the lowest energy system having the same composition.

System	E [hartree]	E [hartree]	ΔE_{rel} [kcal/mol]	ΔE_{rel} [kcal/mol]
	only PCM	with 20 H_2O/PCM	only PCM	with 20 H_2O/PCM
**L1_MS**	-3508.88566935	-5037.16361740	0.0	0.0
**L1_O1**	-3508.83688489 [a]	-5037.16207096	30.6	1.0
**L1_O2**	-3508.84455354 [a]	-5037.13927320	25.8	15.3
**L1:BSF F**	-4993.26739283	-6521.50044149	0.0	0.0
**L1:BSF G**	-4993.24727154	-6521.48635787	12.6	8.8
**L1:CCNU C**	-4636.09847825	-6164.34929533	0.0	0.0
**L1:CCNU G**	-4636.07129514	-6164.33806136	17.1	7.0

[a] Energies from single point calculations performed for the structures of L1 taken from the G configurations of the complexes L1:BSF and L1:CCNU obtained in water described with the PCM.

Table S8. The deformation energies ($E_{def\_vs\_PCM}$) of L1, L1:BSF and L1:CCNU for the structures shown in Figures S20 and S21, resulting from the interaction with 20 water molecules included explicitly, the relative deformation energy (E_{def_rel}) of L1, L1:BSF and L1:CCNU with respect to their structures in the lowest energy complex with 20 H_2O and the interaction energy (E_{int}) of each molecule with the cluster of 20 H_2O. The energies were calculated as:

$$E_{\mathrm{def}\_vs\_PCM}\ MOL = E^{SP}_{MOL\ in\ MOL\_20H_2O} - E^{OPT\ or\ SP(*)}_{MOL\ iwith\ PCM},$$

$$E_{\mathrm{def}\_rel}\ MOL\_20H_2O = E^{OPT}_{MOL(G)\_20H_2O} - E^{OPT}_{MOL(most\ stable)\_20H_2O}\ ,$$

$$E_{\mathrm{def}\_rel}\ 20H_2O = E^{SP}_{20H_2O\ in\ MOL\_20H_2O} - E^{SP}_{20H_2O\ in\ MOL(most\ stable)\_20H_2O}\ ,$$

$$E_{\mathrm{int}}\ with\ 20H_2O = E^{OPT}_{MOL\_20H_2O} - E^{SP}_{MOL\ in\ MOL\_20H_2O} - E^{SP}_{20H_2O\ in\ MOL\_20H_2O}\ .$$

where MOL denotes L1, L1:BSF or L1:CCNU, MOL_20H_2O is the system containing 20 water molecules, SP is the energy obtained from single point calculations performed for the geometry of MOL or 20H_2O taken from the optimized system containing 20 H_2O). All values are in kcal/mol.

System	$E_{def\_vs\_PCM}$ L1	E_{def_rel} L1_20H_2O	E_{def_rel} 20H_2O	E_{int} with 20H_2O
**L1_MS (most stable)**	8.9	0.0	0.0	-123.7
**L1_O1 (from L1:BSF G)**	11.9	33.6	0.5	-156.8
**L1_O2 (from L1:CCNU G)**	9.9	26.8	-1.5	-133.7
	$E_{def\_vs\_PCM}$ L1:DRUG	E_{def_rel} L1:DRUG_20H_2O		
**L1:BSF F**	8.2	0.0	0.0	-137.3
**L1:BSF G**	6.8	11.2	-15.4	-124.4
**L1:CCNU C**	14.1	0.0	0.0	-134.0
**L1:CCNU G**	10.8	13.8	-14.9	-125.8

2.5. Cartesian coordinates for the most stable structures

Table S9. The cartesian coordinates [Å] for the most stable structures shown in the main article.

	Atom no. (b)	Atom	L1_MS			L1:BSF F			L1:CCNU C		
			x	y	z	x	y	z	x	y	z
L1	1	C	-5.63255	1.65592	-2.49513	5.65009	-0.69369	-3.05148	5.33268	-3.77585	1.00364
	2	C	-5.36307	0.17489	-2.68891	5.24031	0.61712	-2.40509	4.97156	-3.72263	-0.47002
	3	O	-5.83356	-0.51988	-1.55023	5.75958	0.64426	-1.09312	5.78445	-2.74932	-1.08973
	4	C	-5.44482	-1.88076	-1.54934	5.26461	1.73071	-0.33862	5.38751	-2.47852	-2.41812
	5	C	-5.90197	-2.53374	-0.26891	5.78938	1.63298	1.07225	6.25447	-1.38075	-2.98332
	6	O	-5.24125	-1.91978	0.82736	5.28473	0.44897	1.66917	6.04929	-0.19578	-2.23085
	7	C	-5.32600	-2.66179	2.03038	5.45109	0.41094	3.07368	6.57788	0.96223	-2.84964
	8	C	-4.24981	-3.75167	2.11026	4.31642	1.14123	3.80184	5.59706	1.56694	-3.86176
	9	H	-5.31363	2.19854	-3.38855	5.28675	-0.71714	-4.08130	4.71967	-4.53271	1.49813
	10	H	-6.70264	1.82077	-2.35160	6.73916	-0.77399	-3.06345	6.38373	-4.05022	1.11632
	11	H	-4.28544	0.00632	-2.80734	4.14600	0.68715	-2.37885	3.91373	-3.45496	-0.57865
	12	H	-5.88355	-0.17526	-3.59359	5.63658	1.45690	-2.99747	5.13632	-4.71290	-0.92297
	13	H	-4.35207	-1.95924	-1.62910	4.16671	1.71432	-0.31779	4.33778	-2.15907	-2.44511
	14	H	-5.90149	-2.40789	-2.40159	5.59371	2.68686	-0.77626	5.49851	-3.37727	-3.04528
	15	H	-5.64619	-3.59892	-0.32301	5.45015	2.51721	1.62356	5.96681	-1.22662	-4.02988
	16	H	-6.98992	-2.42780	-0.14662	6.88879	1.60517	1.07185	7.31551	-1.66651	-2.93734
	17	H	-6.32044	-3.11533	2.14234	6.41912	0.83877	3.36791	7.53456	0.74292	-3.34298
	18	H	-5.19587	-1.94579	2.84952	5.45692	-0.64673	3.36044	6.77764	1.68296	-2.04864
	19	H	-4.45135	-4.54954	1.39608	4.38437	2.21699	3.64334	5.49872	0.92396	-4.73597
	20	H	-4.25254	-4.18313	3.11640	4.39414	0.94658	4.87599	5.97490	2.53962	-4.19173
	21	O	-0.11550	-2.21766	-4.27881	-0.00956	2.79908	-2.08533	-0.36473	-2.91275	-2.68252
	22	C	0.26386	-1.17625	-3.39103	-0.42157	1.44842	-2.02869	-0.49466	-2.15653	-1.49290
	23	C	1.05956	-1.75282	-2.22484	-1.19712	1.17507	-0.74102	-0.84623	-0.71136	-1.83096
	24	O	0.21399	-2.61546	-1.45662	-0.40223	1.47346	0.40305	0.18024	-0.11741	-2.62029
	25	C	-1.10693	-2.16563	-1.15563	0.99275	1.22087	0.32449	1.52245	-0.47636	-2.34377
	26	C	-1.26342	-0.66578	-1.41692	1.28179	0.12750	-0.70925	1.66844	-0.97822	-0.90632
	27	C	-0.96847	-0.38383	-2.89117	0.85973	0.59917	-2.10180	0.83931	-2.25058	-0.73077
	28	O	-0.76222	0.99127	-3.13632	0.65861	-0.47171	-2.99679	0.56485	-2.52640	0.62551
	29	O	-2.58433	-0.23612	-1.15867	2.65206	-0.20374	-0.76272	3.00674	-1.28396	-0.58590
	30	C	1.74977	-0.66414	-1.38351	-1.75935	-0.25723	-0.68529	-1.15982	0.11344	-0.57349
	31	H	0.89607	-0.48707	-3.95971	-1.05598	1.17864	-2.88474	-1.27140	-2.57240	-0.83748
	32	H	1.82960	-2.42550	-2.61601	-2.03972	1.87374	-0.68573	-1.72662	-0.68780	-2.48205
	33	H	-1.83030	-2.70992	-1.77399	1.53329	2.13985	0.07109	1.86618	-1.25117	-3.03776
	34	H	-0.56645	-0.11961	-0.76485	0.69286	-0.75700	-0.41801	1.30004	-0.18239	-0.24027
	35	H	-1.83636	-0.74956	-3.46008	1.65912	1.26342	-2.46300	1.41351	-3.06818	-1.19227
	36	H	-1.60542	1.46311	-2.97984	1.53632	-0.85584	-3.19197	1.41435	-2.72976	1.06901
	37	H	-2.82590	-0.43761	-0.24103	2.96999	-0.41522	0.12855	3.56730	-0.51405	-0.77054
	38	H	1.28235	0.31300	-1.52982	-1.22171	-0.92610	-1.36378	-0.65570	-0.29278	0.30783
	39	H	1.66680	-0.93830	-0.32327	-1.63367	-0.62407	0.34256	-0.80649	1.13859	-0.74011
	40	O	2.41608	3.33473	0.96108	-1.88169	-4.73824	-0.66495	-0.67705	2.34700	3.51234
	41	C	1.01740	3.46480	0.81322	-0.49896	-4.66403	-0.93787	0.54094	1.63437	3.48749
	42	C	0.27585	2.15271	1.06342	0.21757	-3.65273	-0.04836	1.21382	1.66956	2.11804
	43	O	-1.12314	2.40172	0.96631	1.60692	-3.69208	-0.34431	2.47107	1.01054	2.21635
	44	C	-1.49534	2.80042	-0.33688	1.86315	-3.28221	-1.67180	2.33345	-0.35544	2.55052
	45	C	-0.84817	4.13930	-0.70951	1.24348	-4.25174	-2.68466	1.66698	-0.54764	3.91741
	46	C	0.66186	4.00210	-0.57060	-0.25178	-4.33210	-2.40639	0.33410	0.18418	3.91254
	47	O	1.26344	5.26458	-0.78474	-0.83970	-5.31110	-3.23467	-0.24761	0.12219	5.19701
	48	O	-1.18416	4.52169	-2.02986	1.46514	-3.82055	-4.00984	1.46597	-1.91778	4.18845
	49	C	0.56582	1.54826	2.43596	0.01261	-3.91988	1.43991	1.43358	3.09079	1.60111
	50	H	0.69052	4.19052	1.57032	-0.08006	-5.65749	-0.72752	1.21002	2.12431	4.20787

51	H	0.56976	1.41508	0.29725	-0.17470	-2.64442	-0.26590	0.57627	1.13835	1.39216
52	H	-1.16403	2.03995	-1.05784	1.42095	-2.29217	-1.83707	1.70829	-0.86112	1.80119
53	H	-1.20443	4.90487	-0.00396	1.69053	-5.24576	-2.52772	2.31724	-0.09793	4.68403
54	H	1.01727	3.27982	-1.32733	-0.68621	-3.33637	-2.61177	-0.32102	-0.30361	3.16644
55	H	2.19062	5.18241	-0.51849	-1.72400	-5.45993	-2.86948	-0.96947	0.76772	5.18439
56	H	-1.82310	3.88572	-2.40115	2.06517	-3.05382	-3.99548	1.93377	-2.44764	3.51883
57	H	-0.29148	0.94290	2.74078	0.82469	-3.46088	2.00895	2.28383	3.10101	0.91523
58	N	-4.94173	2.19654	-1.32338	5.12876	-1.85300	-2.32809	5.13931	-2.48649	1.66518
59	N	-2.92035	-3.22244	1.82539	3.00524	0.70832	3.33447	4.26682	1.74125	-3.29180
60	C	-5.71659	2.54506	-0.13138	6.03692	-2.67151	-1.52543	6.30633	-1.68334	2.03039
61	C	-5.74104	1.52232	0.99455	6.05919	-2.39641	-0.02828	6.68802	-0.55346	1.08358
62	O	-4.47070	1.50051	1.64262	4.85619	-2.88227	0.56291	5.74395	0.50849	1.20310
63	C	-4.54435	1.38474	3.05724	5.04776	-3.60053	1.77220	6.32250	1.80142	1.29561
64	C	-3.14273	1.22551	3.62369	3.69346	-3.94081	2.37263	5.21916	2.84675	1.27937
65	O	-2.72767	-0.12692	3.72578	3.17251	-2.92393	3.20921	4.82993	3.23746	-0.02452
66	C	-2.66401	-0.80226	2.48020	2.97421	-1.68348	2.55394	4.31617	2.17695	-0.81179
67	C	-2.36151	-2.27497	2.78816	2.60744	-0.66407	3.64015	4.11825	2.73267	-2.22825
68	C	-3.62346	2.48185	-1.44815	3.83074	-2.18259	-2.53308	3.87590	-2.14800	2.02299
69	C	-2.52294	-3.23946	0.51401	2.50718	1.37501	2.24177	3.43603	0.64836	-3.36781
70	H	-6.74473	2.71065	-0.46163	7.04270	-2.50739	-1.91877	7.15282	-2.37037	2.10048
71	H	-5.34788	3.49884	0.26300	5.79678	-3.72838	-1.68717	6.14731	-1.26512	3.03077
72	H	-5.97308	0.51890	0.62032	6.15391	-1.32562	0.17719	6.71358	-0.89568	0.04460
73	H	-6.51122	1.83950	1.70954	6.91961	-2.93172	0.39278	7.68229	-0.19707	1.38090
74	H	-5.15796	0.51515	3.33609	5.62563	-2.99841	2.48873	7.00524	1.97691	0.45133
75	H	-5.01782	2.28671	3.46801	5.61123	-4.52118	1.56677	6.90173	1.87873	2.22614
76	H	-3.11412	1.61606	4.64328	3.79357	-4.82200	3.01100	5.57991	3.75760	1.76352
77	H	-2.44226	1.80058	3.00032	2.99707	-4.17289	1.55444	4.36074	2.45955	1.84669
78	H	-1.87800	-0.34147	1.86113	2.17792	-1.79042	1.80070	3.37396	1.81670	-0.37067
79	H	-3.61531	-0.72380	1.94178	3.88508	-1.35925	2.03931	5.01444	1.33419	-0.84265
80	H	-2.81619	-2.50576	3.75548	3.14403	-0.95440	4.54737	4.89227	3.48791	-2.38890
81	H	-1.28443	-2.42988	2.89558	1.53929	-0.71438	3.86592	3.15206	3.23891	-2.30859
82	N	-2.94563	2.86175	-0.32050	3.30839	-3.19803	-1.77815	3.68172	-0.88824	2.51510
83	N	-1.41882	-2.47319	0.20697	1.43274	0.78724	1.61997	2.33963	0.68261	-2.54008
84	O	-3.11029	-3.89907	-0.35015	2.99131	2.43610	1.83870	3.65568	-0.30367	-4.11854
85	O	-3.04759	2.45486	-2.56289	3.14849	-1.62081	-3.42104	2.93443	-2.97291	1.96452
86	H	-3.33754	2.54811	0.56942	3.76462	-3.36285	-0.87966	4.36726	-0.18420	2.23703
87	H	-0.84538	-2.02659	0.92302	0.93456	-0.00917	2.01801	2.03932	1.52471	-2.05010
88	H	0.71779	2.33693	3.17823	0.01542	-4.99572	1.63805	1.65344	3.76763	2.43190
89	H	-0.34625	-2.99949	-3.75731	-0.78430	3.38526	-2.03702	-1.15632	-2.75142	-3.21579
90	C	4.06822	-0.72345	1.73878	-3.72874	-2.06375	1.94783	-2.28452	3.79597	-0.25545
91	C	4.84080	-0.30440	2.99285	-4.40773	-3.19731	2.72381	-2.57491	5.18913	0.30667
92	O	4.49800	1.02441	3.38676	-3.93129	-4.46655	2.28320	-1.99748	5.34197	1.60171
93	C	5.46939	2.01221	3.07370	-4.84645	-5.20291	1.49041	-2.92962	5.32568	2.66886
94	C	5.65257	2.28143	1.59170	-5.14473	-4.61292	0.12452	-3.58845	3.98278	2.92210
95	O	6.33774	1.19911	0.97974	-5.91090	-3.42673	0.26193	-4.48243	3.67133	1.86726
96	C	6.94032	1.53008	-0.25673	-6.64791	-3.09651	-0.89683	-5.43581	2.68821	2.21163
97	C	5.93746	1.79673	-1.37467	-5.77275	-2.62293	-2.05294	-4.84477	1.28840	2.33754
98	H	4.65534	-1.44732	1.16927	-4.41239	-1.22069	1.82902	-3.09120	3.48406	-0.92346
99	H	3.93088	0.14712	1.08750	-3.49074	-2.41655	0.93678	-2.26086	3.07512	0.56866
100	H	5.91698	-0.36947	2.81156	-5.49213	-3.14466	2.60083	-3.65338	5.35760	0.35883
101	H	4.57841	-0.96828	3.82379	-4.16586	-3.10455	3.78870	-2.12833	5.94725	-0.34665
102	H	6.43524	1.73428	3.51702	-5.78992	-5.33256	2.03811	-3.70695	6.08230	2.49638
103	H	5.11719	2.93111	3.54962	-4.39044	-6.18593	1.34585	-2.36583	5.60422	3.56312
104	H	6.24343	3.20348	1.48373	-5.71644	-5.36303	-0.44317	-4.13891	4.04903	3.87289
105	H	4.67493	2.44067	1.11889	-4.21255	-4.41315	-0.41547	-2.82015	3.20804	3.02640
106	H	7.58001	2.41698	-0.13769	-7.24252	-3.96027	-1.22913	-5.92898	2.95133	3.15876
107	H	7.58235	0.68137	-0.51461	-7.34288	-2.30312	-0.60094	-6.19369	2.70667	1.42120
108	H	5.24469	2.58993	-1.09316	-5.02877	-3.37748	-2.30882	-4.01985	1.27740	3.05046
109	H	6.48637	2.13428	-2.26166	-6.40559	-2.46345	-2.93281	-5.61899	0.61003	2.71291

	110	N	2.77303	-1.34244	2.00345	-2.51990	-1.54568	2.57716	-1.04417	3.70555	-1.01655
	111	N	5.15878	0.61330	-1.72109	-5.07097	-1.38538	-1.74583	-4.34925	0.77376	1.06585
	112	C	2.70907	-2.79571	2.11815	-2.62897	-0.39099	3.45904	-1.11454	3.88891	-2.46036
	113	C	2.31111	-3.49844	0.82581	-2.40200	0.93929	2.75367	-1.25243	2.58251	-3.23030
	114	O	3.18002	-3.23225	-0.26442	-3.31562	1.18152	1.69430	-2.38389	1.81095	-2.85207
	115	C	4.43814	-3.87394	-0.18917	-4.62720	1.49537	2.12112	-3.61413	2.26445	-3.37782
	116	C	5.01975	-3.91510	-1.59022	-5.31314	2.24745	0.99762	-4.56632	1.08265	-3.42851
	117	O	5.02447	-2.63976	-2.21038	-5.21971	1.56694	-0.24166	-4.68856	0.41719	-2.18098
	118	C	6.06809	-1.76184	-1.80911	-6.13206	0.49272	-0.40922	-5.45476	1.10056	-1.19659
	119	C	5.83356	-0.41835	-2.50680	-5.83875	-0.14697	-1.77269	-5.34282	0.30195	0.10420
	120	C	1.61891	-0.63094	2.16167	-1.28149	-2.08783	2.37524	0.16613	3.40961	-0.45255
	121	C	3.81942	0.59263	-1.45476	-3.73655	-1.42662	-1.45724	-3.00638	0.63857	0.88290
	122	H	1.97303	-3.06163	2.88239	-1.88369	-0.48158	4.25346	-0.20237	4.38524	-2.80251
	123	H	3.68626	-3.14163	2.47183	-3.61747	-0.42504	3.93020	-1.95344	4.56263	-2.66652
	124	H	2.26426	-4.58236	1.00956	-2.46168	1.75459	3.49128	-1.29100	2.79971	-4.30784
	125	H	1.32613	-3.15264	0.50859	-1.41143	0.94242	2.29718	-0.38444	1.95648	-3.02437
	126	H	4.32922	-4.90520	0.17616	-4.60526	2.13855	3.01256	-3.48767	2.64723	-4.40022
	127	H	5.11105	-3.34854	0.50394	-5.18468	0.58586	2.38888	-4.02785	3.08487	-2.77280
	128	H	4.39057	-4.54474	-2.22435	-4.82147	3.21002	0.84088	-4.16902	0.32839	-4.11304
	129	H	6.03406	-4.33515	-1.56601	-6.36469	2.43048	1.25738	-5.55236	1.40994	-3.78516
	130	H	6.07880	-1.62250	-0.71879	-6.02295	-0.26062	0.38524	-5.08672	2.12390	-1.03869
	131	H	7.03138	-2.18830	-2.11652	-7.15768	0.88261	-0.37494	-6.49956	1.15083	-1.52740
	132	H	5.27313	-0.61031	-3.43024	-5.31867	0.60150	-2.38217	-5.14734	-0.74279	-0.16061
	133	H	6.79847	0.00052	-2.79729	-6.77990	-0.37616	-2.27531	-6.30569	0.31959	0.61733
	134	N	1.73904	0.70142	2.40407	-1.24008	-3.35769	1.89358	0.26874	3.57508	0.89364
	135	N	3.13692	-0.53721	-1.78864	-3.15293	-0.25720	-1.08267	-2.58959	0.08067	-0.30083
	136	O	3.23350	1.57190	-0.94769	-3.07688	-2.47872	-1.56951	-2.18331	0.95465	1.75764
	137	O	0.50232	-1.18366	2.08891	-0.24432	-1.45136	2.63770	1.12617	3.01901	-1.14193
	138	H	3.65040	-1.41441	-1.87686	-3.72586	0.47982	-0.67335	-3.17746	0.23272	-1.12362
	139	H	2.64474	1.09928	2.63392	-2.09261	-3.89360	1.75967	-0.48179	4.01196	1.41907
	140	H	2.73237	2.66433	0.32051	-2.27364	-3.86532	-0.87627	-1.26678	1.92018	2.85975
BSF	141	C	-3.49796	0.21722	2.83054	1.46630	5.15370	1.53460			
	142	C	-3.76339	1.67906	3.15058	0.08770	5.59461	1.98976			
	143	C	-4.31720	2.49006	1.97648	-0.93658	4.45951	2.09152			
	144	C	-3.42828	2.51123	0.74844	-1.60156	4.09040	0.78254			
	145	O	-2.15135	3.06879	1.16526	-2.34499	5.26982	0.33593			
	146	O	-2.30116	0.04563	2.01496	1.46394	4.58640	0.19257			
	147	S	-0.98274	3.03272	0.07191	-3.17238	5.13291	-1.02275			
	148	S	-0.88789	-0.06397	2.77178	1.38389	5.59966	-1.04513			
	149	O	-0.66694	1.64460	-0.25322	-2.58144	4.07879	-1.84865			
	150	O	-1.34748	3.90490	-1.04084	-4.58614	5.00415	-0.69860			
	151	O	-0.32222	1.26671	2.96424	0.01396	5.62496	-1.54831			
	152	O	-1.05965	-0.89257	3.96176	1.98492	6.87074	-0.66154			
	153	C	0.30567	3.75638	1.03926	-2.87876	6.72497	-1.73107			
	154	C	0.01624	-0.93260	1.52619	2.42242	4.73424	-2.18343			
	155	H	-4.29128	-0.21648	2.21729	1.85286	4.32813	2.13277			
	156	H	-3.38387	-0.38384	3.73511	2.17552	5.98399	1.55140			
	157	H	-4.49509	1.70543	3.96493	0.22693	6.04380	2.97887			
	158	H	-2.84462	2.13573	3.53353	-0.29174	6.39144	1.33887			
	159	H	-5.28327	2.07624	1.66435	-0.45127	3.54515	2.45700			
	160	H	-4.49937	3.51988	2.29952	-1.71488	4.72321	2.81467			
	161	H	-3.84647	3.15156	-0.03259	-2.30126	3.26079	0.92998			
	162	H	-3.25176	1.50763	0.35216	-0.87453	3.83196	0.01454			
	163	H	-0.01303	4.75528	1.33449	-3.22569	7.47386	-1.01972			
	164	H	0.47112	3.10486	1.89662	-1.81064	6.81148	-1.92099			
	165	H	1.18330	3.80115	0.39349	-3.46047	6.77135	-2.65188			
	166	H	-0.46279	-1.89804	1.37020	2.44316	5.32135	-3.10188			
	167	H	1.02870	-1.05274	1.91346	1.96477	3.75889	-2.35000			
	168	H	0.00560	-0.31439	0.62888	3.41594	4.65846	-1.74398			

CCNU	141	C	3.56292	1.58195	-0.09832				-1.99864	-2.73631	3.78788
	142	C	2.14472	1.07683	-0.37578				-2.53641	-2.22607	2.44858
	143	C	1.90270	-0.26844	0.31568				-3.22706	-3.34399	1.66790
	144	C	4.60657	0.54758	-0.52803				-1.06609	-3.93144	3.58338
	145	C	4.35632	-0.80033	0.15377				-1.77677	-5.05306	2.82418
	146	C	2.93653	-1.30718	-0.11572				-2.28926	-4.54100	1.47700
	147	N	0.55886	-0.77395	0.03636				-3.65677	-2.80975	0.37399
	148	C	-0.50378	-0.32202	0.71479				-4.51538	-3.47531	-0.40986
	149	N	-1.77577	-0.85033	0.29719				-4.59038	-2.97835	-1.76665
	150	C	-3.01734	-0.36890	0.89645				-5.66010	-3.39112	-2.66704
	151	C	-3.81649	0.51334	-0.04945				-6.59644	-2.23662	-2.97450
	152	N	-1.76912	-1.80026	-0.62955				-3.53219	-2.32162	-2.21635
	153	O	-2.86542	-2.22130	-0.94424				-3.55490	-2.03926	-3.40159
	154	Cl	-2.86934	1.96328	-0.54747				-7.53212	-1.76875	-1.50620
	155	O	-0.47095	0.47421	1.64021				-5.21392	-4.41810	-0.08707
	156	H	3.67185	1.78170	0.97664				-2.84341	-3.03807	4.42272
	157	H	3.72742	2.53231	-0.61615				-1.47255	-1.93016	4.30992
	158	H	2.00402	0.93924	-1.45694				-1.69561	-1.85245	1.84267
	159	H	1.39569	1.79989	-0.03528				-3.23312	-1.39321	2.59790
	160	H	1.96833	-0.12620	1.40115				-4.12557	-3.66991	2.20696
	161	H	4.55547	0.41562	-1.61765				-0.19489	-3.59703	3.00641
	162	H	5.61501	0.90581	-0.29603				-0.69459	-4.29092	4.54871
	163	H	4.49818	-0.68929	1.23740				-2.62077	-5.42370	3.42218
	164	H	5.08356	-1.54393	-0.18727				-1.10319	-5.90135	2.66602
	165	H	2.81732	-1.50152	-1.19158				-1.43454	-4.20608	0.87448
	166	H	2.75360	-2.25007	0.41068				-2.81271	-5.32787	0.92382
	167	H	0.41581	-1.40141	-0.74450				-3.16570	-1.99892	0.00196
	168	H	-3.62616	-1.23865	1.15828				-5.20076	-3.73119	-3.59870
	169	H	-2.73800	0.17042	1.79925				-6.17858	-4.21900	-2.18694
	170	H	-4.71206	0.87324	0.45522				-7.31923	-2.52363	-3.73632
	171	H	-4.09094	-0.01127	-0.96227				-6.04255	-1.35106	-3.28378

References

[SR1] *HyperChem(TM) Professional*, version 8.0.10. 1115 NW 4th Street, Gainesville, Florida 32601, USA: Hypercube, Inc; 2011.

[SR2] Stewart, J.J.P. *MOPAC2016*, Stewart Computational Chemistry, 2016, http://OpenMOPAC.net (accessed October 15, 2019).

[SR3] Guarnieri, F.; Still, W.C. A Rapidly Convergent Simulation Method: Mixed Monte Carlo/Stochastic Dynamics. *J. Comput. Chem.* **1994**, *15*, 1302−1310.

[SR4] Allouche, R. *Gabedit 2.5.1*, Gabedit - A Graphical User Interface for Computational Chemistry Softwares. *J. Comput. Chem.* **2011**, *32*, 174−182.

[SR5] Frisch, M. J.; Trucks, G. W.; Schlegel, H. B.; Scuseria, G. E.; Robb, M. A.; Cheeseman, J. R.; Scalmani, G.; Barone, V.; Mennucci, B.; Petersson, G. A., et al. *Gaussian 09*, Revision D.01; Gaussian, Inc.: Wallingford, CT, 2013.

[SR6] Zhao, Y.; Truhlar, D. G. The M06 Suite of Density Functionals for Main Group Thermochemistry, Thermochemical Kinetics, Noncovalent Interactions, Excited States, and Transition Elements: Two New Functionals and Systematic Testing of four M06-Class Functionals and 12 Other Functionals. *Theor. Chem. Acc.* **2008**, *120*, 215−241.

[SR7] Grimme, S.; Antony, J.; Ehrlich, S.; Krieg, H. A Consistent and Accurate Ab Initio Parameterization of Density Functional Dispersion Correction (DFT-D) for the 94 Elements H-Pu. J. Chem. Phys. **2010**, *132*, 154104.

[SR8] Tomasi, J.; Mennucci, B.; Cammi, R. Quantum Mechanical Continuum Solvation Models. *Chem. Rev.* **2005**, *105*, 2999−3093.

[SR9] Frisch, M. J.; Trucks, G. W.; Schlegel, H. B.; Scuseria, G. E.; Robb, M. A.; Cheeseman, J. R.; Scalmani, G.; Barone, V.; Petersson, G. A.; Nakatsuji, H.; et al. *Gaussian 16*, Revision C.01. Gaussian, Inc., Wallingford CT, 2016.

[SR10] Klamt, A.; Schüümann, G. COSMO: a New Approach to Dielectric Screening in Solvents with Explicit Expressions for the Screening Energy and Its Gradient. *J. Chem. Soc., Perkin Trans. 2* **1993**, 799–805.

[SR11] Tantillo, D. J. Chemical Shift Repository, http://cheschirenmr.info/Instructions.htm (accessed February 1, 2023).
